# Fast and accurate detection of spread source in large complex networks

**DOI:** 10.1038/s41598-018-20546-3

**Published:** 2018-02-06

**Authors:** Robert Paluch, Xiaoyan Lu, Krzysztof Suchecki, Bolesław K. Szymański, Janusz A. Hołyst

**Affiliations:** 1 Center of Excellence for Complex Systems Research, Faculty of Physics, Warsaw University of Technology, Koszykowa 75, 00662 Warsaw, Poland; 2 Social Cognitive Networks Academic Research Center, Rensselaer Polytechnic Institute, 110 8th Street, Troy, NY 12180-3590 USA; 3 The ENGINE Centre, Wroclaw University of Science and Technology, Wyb. Wyspianskiego 27, 50-370 Wroclaw, Poland; 4 ITMO University, 49 Kronverkskiy av., 197101 Saint Petersburg, Russia

## Abstract

Spread over complex networks is a ubiquitous process with increasingly wide applications. Locating spread sources is often important, e.g. finding the patient one in epidemics, or source of rumor spreading in social network. Pinto, Thiran and Vetterli introduced an algorithm (PTVA) to solve the important case of this problem in which a limited set of nodes act as observers and report times at which the spread reached them. PTVA uses all observers to find a solution. Here we propose a new approach in which observers with low quality information (i.e. with large spread encounter times) are ignored and potential sources are selected based on the likelihood gradient from high quality observers. The original complexity of PTVA is *O*(*N*^*α*^), where *α* ∈ (3,4) depends on the network topology and number of observers (*N* denotes the number of nodes in the network). Our Gradient Maximum Likelihood Algorithm (GMLA) reduces this complexity to *O* (*N*^2^log (*N*)). Extensive numerical tests performed on synthetic networks and real Gnutella network with limitation that id’s of spreaders are unknown to observers demonstrate that for scale-free networks with such limitation GMLA yields higher quality localization results than PTVA does.

## Introduction

We live in the networked society. Every second we interact with many networks from which we collect, process and transmit a huge amount of information, which increases exponentially each year^1–4^. Increasing interconnectivity of the world exposes us to world-wide range of pathogens, viruses both physical and virtual, misinformation and rumors with often grievous consequences^5–8^. A good example is a fake tweet about explosion in White House in 2013, which caused $130 billion loss on the stock market^9^. Another example is the United States presidential election of 2016 when many rumors or fake news became viral on Facebook or Twitter and might have affected elections^10^. Many papers seek finding best conditions for spreading^11–16^ or sets of optimal spreaders^17–20^ but here we investigate an inverse problem. It became clear that one of the major challenges facing network and data scientists is to develop effective methods for detecting and suppressing spread of dangerous viruses, pathogens, misinformation or gossips. The basic component of such a system is undoubtedly a fast algorithm finding a source of such spread. The first widely discussed research on this subject has been done by Shah and Zaman^21^ and Pinto, Thiran and Vetterli^22^. In social networks, Shah and Zaman introduced *rumor centrality* of a node as the number of distinct ways a rumor can spread in the network starting from that node. They showed that the node with maximum *rumor centrality* is the Maximum Likelihood Estimator of the rumor source if the underlying graph is a regular tree. They studied also the detection performance for irregular geometric trees, small-word networks and scale-free networks. This method assumes that we know all the connections between nodes and additionally the infection states of all nodes. Pinto *et al*. relaxed some of these constraints since their algorithm requires information about state of not every node, but only about some fraction of nodes called *observers*. A further description of this algorithm is given in the next section and in Supplementary Information. After these two publications, the topic of the source detection became popular and many other variants of this problem have been studied. We can distinguish two main approaches to this issue: the snapshot-based^21,23–25^ and the detector-based^22,26,27^ source detection. The first one requires the snapshot of an entire network at a certain time instance, the second needs to monitor only a small subset of nodes but all the time. Regardless of the above division, researchers considered also different epidemic models^25,28^, spreading at weighted or time-varying graphs^29–32^ and multi-source detection problems^33,34^. In 2014 Jiang *et al*. described state-of-the-art and conducted comparative studies^35^. One of their conclusions is that current methods are too computationally expensive and they can not be use for a quick identification of the propagation source. The main goal of our research was finding the method which executes in reasonable time on large complex networks and delivers high quality of localization results at the same time.

## Results

Before demonstrating our main results, we present a brief description of Pinto-Thiran-Vetterli algorithm (PTVA)^22^. Then, we introduce our approach in which observers with low quality information (i.e. with large spread encounter times) are ignored and potential sources are selected based on the likelihood gradient from high quality observers. In order to measure the performance of the algorithms we use three different quality of localization measures: the accuracy, the rank and the distance error. The accuracy is the empirical probability that a source found by the algorithm is the true source. The rank is the true source position on the nodes list, which is sorted in descending order by likelihood of being the source. The distance error is the shortest path distance between the real source and the source found by the algorithm. Details on these measures can be found in the section Methods.

### Pinto-Thiran-Vetterli Algorithm

Pinto, Thiran and Vetterli^22^ proposed a general framework for the localization of the spread source in which some of the nodes in network act as observers and report from which neighbor and at what time it received the information. However, in real life the identity of the neighbor that sent the message to the observer is not always available (like in the case of gossip spreading on the public square). For this reason, and for the sake of greater generality and applicability of our studies, we do not require data received by observers to contain the identities of nodes from which the spread came. We refer to tests in which PTVA is applied to such data as Pinto-Thiran-Vetterli Algorithm executed on data with Limited Information (PTVA-LI). This lowering of the requirements on input data increases applicability of the methods but reduces detection accuracy, and yet it does not affect the algorithm’s complexity or speed. Thus, PTVA-LI tests for the speed and complexity are valid also for PTVA.

PTVA calculates the likelihood of each node to be the source (which we call the score, see Eq. 1 in Sup. Inf. Section S.1) using the reported times (observed delays) from all available observers. For this purpose, PTVA assumes information spreads through the network along the shortest paths and therefore uses breadth-first search (BFS) tree in place of the actual but unknown propagation tree. The method also assumes that the propagation times *θ*_*i*_ for each edge are i.i.d Gaussian random variables, for which the mean *μ* and the variance *σ*^2^ are known. The algorithm’s complexity for arbitrary graphs is *O* (*N*(*K*^3^ + *N*^2^)), where *N* is the size of the network and *K* is the number of observers. If *K* ~ *N*^γ^, PTVA complexity ranges from *O*(*N*^3^) when *γ* ≤ 2/3 to *O*(*N*^4^) when *γ* = 1. For more details on PTVA see Sup. Inf. Section S.1.

**Algorithm 1:**
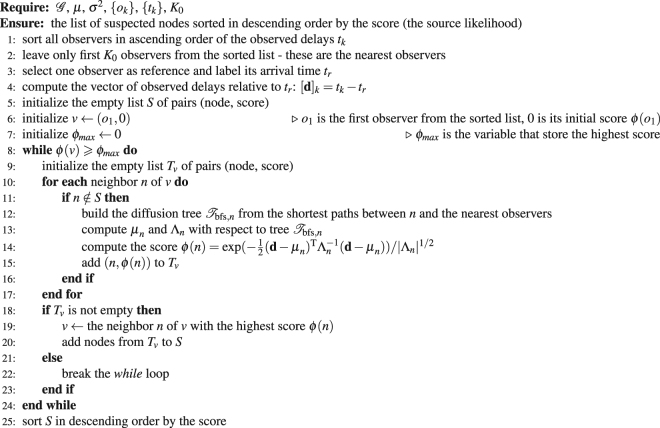
Gradient Maximum Likelihood Algorithm.

### Gradient Maximum Likelihood Algorithm

#### Description

Compared to the framework introduced by PTVA-LI we propose two improvements: a limited number of observers, and a gradient-like selection of suspected nodes. The first idea takes advantage of the fact that observers which are very far from the spread source make very small contribution to the score in comparison to the nearest observers (Fig. S4 in Sup. Inf. Section S.2). On the other hand, those distant observers increase greatly the cost of information processing. Since a distance between any observer *o*_*k*_ and the true source should increase (in average) with the arrival time *t*_*k*_, we can use only a small number *K*_0_ ≪ *K* of the nearest observers and drastically shorten the time needed for computing the score. The limited number of observers was used in earlier work^22,36^ where the search algorithm was run before all *K* observers get infected in order to limit the outbreak. In contrast, here we focus on the optimization of the algorithm’s complexity for large complex networks.

**Figure 1:**
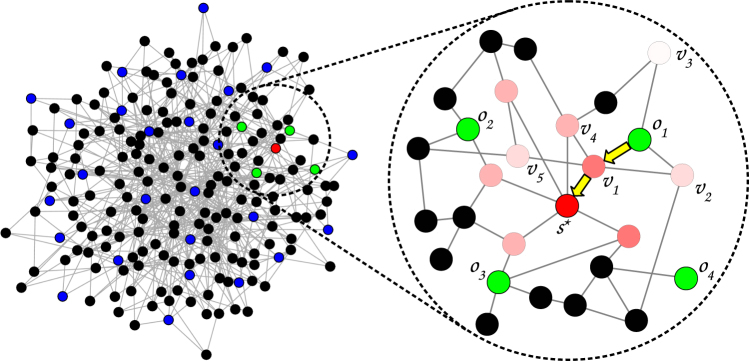
The visualization of GMLA. The left picture presents the whole graph. The red node is the true source. Green nodes mark the *K*_0_ nearest observers with the smallest time delays (in this plot *K*_0_ = 4). The rest of the observers in the network are highlighted in blue. The picture on the right is a zoom of a small area around the nearest observers. In this picture the color corresponds to the score (the likelihood of being the source) of the node (except for the observers which are green). The higher the score of the node is, the darker red is its color. At the beginning the algorithm computes the scores for the neighbors of the nearest observer (in this plot *the observer one* is *o*_1_ and its neighbors are *v*_1_, *v*_2_, and *v*_3_). Afterwards GMLA selects the neighbor with the highest score (*v*_1_ in this case) and starts computing the scores for its neighbors (*o*_1_, *v*_4_, *v*_5_ and s^*^). During this step there is no need for estimating the likelihood for the node *v*_2_ since it was done in the previous step. All the scores which are computed are stored in the list. Since s^*^ has the highest score among the neighbors of *v*_1_, in the next step GMLA will compute the scores for it neighbors. None of the neighbors of s^*^ has higher score than s^*^, therefore the algorithm stops here. The node s^*^ is the source according to GMLA because it has the highest score from all tested (suspected) nodes. The nodes not visited by the algorithm are black since their scores are undefined (their scores are not computed).

The second idea introduces a procedure of the nodes selection for the score calculation. It is very likely that the spread source is in close proximity to the observer which has the smallest time at which the spread was observed (the *observer one*). The procedure starts by calculating scores of the nearest neighbors of the *observer one* and then selects a neighbor with the highest score. Next, the algorithm jumps into this node and calculates scores for its nearest neighbors in order to find the one which has a score greater than or equal to the current maximum. The process is gradient-like and it is continued until all neighbors have a score lower than the current maximum (see Fig. 1). Each calculated score is remembered (along with the node) which allows the algorithm to avoid double-calculation and to prepare a ranking of nodes suspected to be the source. The number of suspected nodes *N*_0_ = |*V*_*s*_| depends primarily on the size of the network and the average degree 〈*k*〉. The empirical studies shows that ${N}_{0}\sim \langle k\rangle \,\mathrm{log}(N)$ (Fig. S6 in Sup. Inf. S.2). It is worth noting that the algorithm does not guarantee that the true source s^*^ will be selected for score calculation, i.e. P(s^*^∈*V*_*s*_) < 1 (see Fig. S9a and S10a in Sup. Inf. S.2).

The Gradient Maximum Likelihood Algorithm (GMLA) is summarized in Algorithm 2. ${\mathscr{G}}$ denotes the underlaying graph, *μ* and *σ*^2^ denote the mean and the variance of the random propagation delay associated with one edge, {*o*_*k*_} is the set of observers and {*t*_*k*_} are the times at which they observed the spread. The score of a node is the likelihood that this node is the true source. We denote the score of a node *v* as *ϕ*(*v*). The formulas for *ϕ*(*v*), *μ*_*v*_ and Λ_*v*_ are given by equations (1,3,4) in Sup. Inf.

#### Complexity

Using the symbols *K*_0_ and *N*_0_ we reformulate the time complexity of GMLA as $O({N}_{0}({K}_{0}^{3}+{N}^{2}))$ in the worst case. Assuming ${N}_{0}\sim \,\mathrm{log}(N)$ and *K*_0_ ≪ *N*, which is true for our method, the complexity can be further simplified into *O*(log(*N*)*N*^2^).

#### Fine-tuning and performance

**Figure 2:**
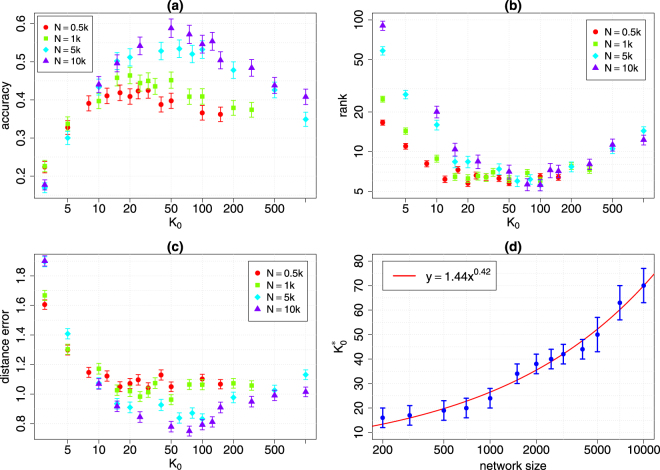
Performance of GMLA versus number of the nearest observers *K*_0_ for various sizes of BA network (*m* = 3). (**a**) The maximum of accuracy is not very sharp but is clearly visible. (**b**,**c**) The minima of rank and distance error are not always compatible with the maximum of accuracy which increases the uncertainty of finding ${K}_{0}^{\ast }$. (**d**) The optimal number of the nearest observers ${K}_{0}^{\ast }$ for various sizes of BA network. The solid line is a nonlinear least squares model $y=b{x}^{a}$ (Gauss-Newton algorithm), where *a* = 0.42 ± 0.04 and *b* = 1.44 ± 0.51 (95% confidence interval).

The number of the nearest observers *K*_0_ is a crucial parameter of GMLA and should be carefully selected. If *K*_0_ is too small, the accuracy of the algorithm decreases. On the other hand, large *K*_0_ increases the time of computation. The optimal number of the nearest observers ${K}_{0}^{\ast }$ is the minimal number of the nearest observers *K*_0_ needed to achieve maximal quality of the spread source localization. We test how ${K}_{0}^{\ast }$ depends on the network size, the average degree and the propagation ratio for Erdös-Rényi (ER) and Barabási-Albert (BA) networks^37^ (see Sup. Inf. Section S.3). No substantial relationship was found between ${K}_{0}^{\ast }$ and the average degree of the network or the propagation ratio (Figs S15–S18 in Sup. Inf. S.3). Figure 2 presents how the number of the nearest observers affects the performance of GMLA for various sizes of BA network with the minimum degree *m* = 3 (*m* is the initial degree of each attached node, thus 〈*k*〉 = 2*m* = 6). It is easy to see a peak of the accuracy and the valleys of the rank and distance error. Figure 2d shows the estimates of ${K}_{0}^{\ast }$ for different sizes of BA network. In the case of Erdös-Rényi network, no peak of the accuracy is observed, but the saturation point is clearly visible (Fig. S13c in Sup. Inf. S.3). This also applies to the rank of the true source and the distance error (Fig. S13d,e). The fact that we can observe the peak of the accuracy for BA networks (not only the saturation point like for ER graphs) has substantial consequences, because it means that taking only *K*_0_ ≪ *K* nearest observers not only shortens the computation time, but it may also improve the quality of the source localization under certain circumstances. As we show further in Discussion, such a circumstance is the occurrence of the hubs in BA network. In the next paragraphs we present a numerical estimation of the complexity of GMLA as well as its performance in terms of the quality of results in comparison to PTVA-LI.

### Tests on synthetic networks

We tested GMLA and PTVA-LI for various sizes of Erdös-Rényi (ER) random graphs and Barabási-Albert (BA) networks. We used Susceptible-Infected model (see details in section Methods) for the spread with the infection rate *β* = 0.5 ($\lambda =\sqrt{2}$). The observers were distributed randomly over a whole network with the density *ρ* = 0.2. In order to maintain a high efficiency of GMLA, we set the number of the nearest observers as a function of the network size ${K}_{0}=0.5\sqrt{N}$ (see Fig. 2d and Fig. S12 in Sup. Inf. S.3) For comparative purposes, we introduce also a baseline method. The baseline method is very naive and according to it, the true source is always the *observer one* (with smallest delay *t*_*k*_). Details on the baseline method are given in section Methods.

**Figure 3:**
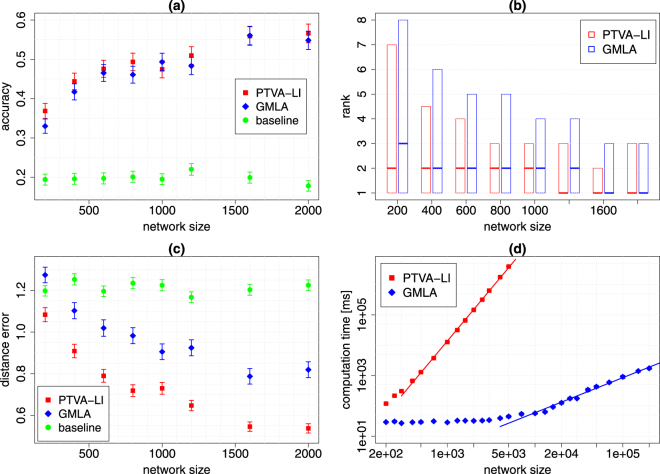
GMLA (blue) versus PTVA-LI (red) and the baseline method (green) on ER network. (**a**) The accuracy of both algorithms is almost the same and it increases with the network size. (**b**) The band inside the box shows the median of the true source rank. The bottom and top of the box show the values of the first and third quartiles. (**c**) The mean distance error for PTVA-LI and GMLA decreases with the network size. (**d**) The mean computation time of a single realization of GMLA is substantially shorter than of PTVA-LI. The solid lines are linear models *ln*(time) = *a* ln(size) + *b*, where *a* = 3.46 ± 0.07 for PTVA-LI and *a* = 1.15 ± 0.09 for GMLA (95% confidence interval). In the figures (**a**–**c**) each point is the result of 1000 realizations. In the figure (**d**) results for PTVA-LI and GMLA are averaged over 26 realizations. Parameters: 〈*k*〉 = 6, $\lambda =\sqrt{2}$, ρ = 0.2, ${K}_{0}=0.5\sqrt{N}$ (details in the text).

**Figure 4:**
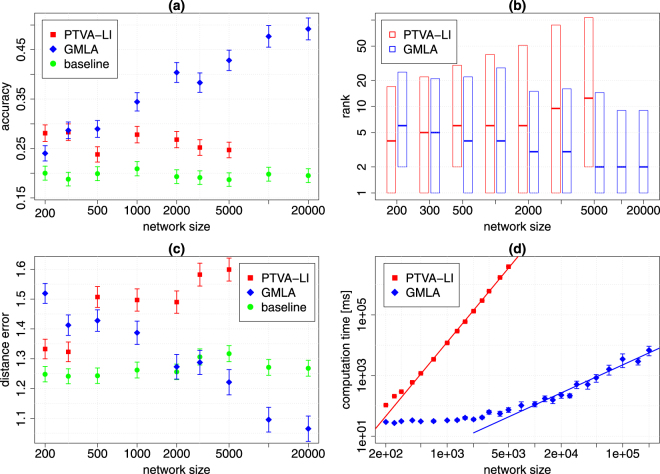
GMLA (blue) versus PTVA-LI (red) and the baseline method (green) on BA network (*m* = 3). (**a**) The accuracy of GMLA increases with the network size and it is much higher than accuracy of PTVA-LI. (**b**) The band inside the box shows the median of the true source rank. The bottom and top of the box show the values of the first and third quartiles. GMLA has much lower rank than PTVA-LI. (**c**) The mean distance error for PTVA-LI increases with the network size in contrast to GMLA, which initially is the highest in the plot, but becomes the lowest for the networks larger that 3000 nodes. (**d**) The mean computation time of a single realization of GMLA is substantially shorter than of PTVA-LI. The solid lines are linear models $\mathrm{ln}({\rm{time}})=a\,\mathrm{ln}({\rm{size}})+b$, where *a* = 3.49 ± 0.08 for PTVA-LI and *a* = 1.32 ± 0.08 for GMLA (95% confidence interval). In the figures (**a**)–(**c**) each point is the result of 1000 realizations. In the figure (**d**) results for PTVA-LI and GMLA are averaged over 26 realizations. Parameters: 〈*k*〉 = 6, $\lambda =\sqrt{2}$, *ρ* = 0.2, ${K}_{0}=0.5\sqrt{N}$ (details in the text).

The most important feature of GMLA is a remarkable reduction of the computation time. Figures 3d and 4d show that the empirical complexity decreases from *O*(*N*^3.46^) to *O*(*N*^1.15^) for ER graph and from *O*(*N*^3.49^) to *O*(*N*^1.32^) for BA network. Furthermore, one can observe an initial difference between GMLA and PTVA-LI computing times for the networks of size 200, which is a factor 4.4 for ER graph and 3.6 for BA network.

The quality of the source localization clearly depends on the network topology. In general, both algorithms achieve better results for ER graphs than BA networks. In the case of ER graphs, the accuracy of both algorithms is almost the same (Fig. 3a), but PTVA-LI is characterized by lower rank and distance error (Fig. 3b,c). On the other hand, for BA networks which are larger than 300 nodes GMLA outperforms PTVA-LI in every test of quality of the results (Fig. 4a–c). Moreover, the advantage of GMLA increases with the size of BA network and is especially high for large networks, for which the computation of PTVA-LI takes too long to collect a large enough statistics.

### Tests on real social network

**Figure 5:**
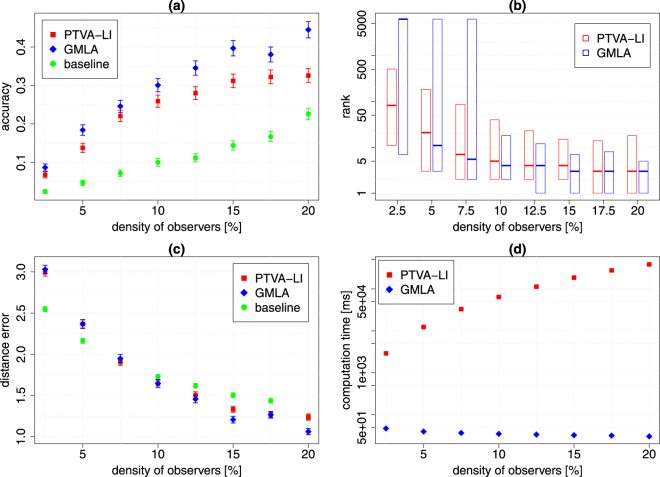
GMLA (blue) versus PTVA-LI (red) and the baseline method (green) on Gnutella network. (**a**) GMLA achieves higher accuracy than PTVA-LI and the baseline method. (**b**) The band inside the box shows the median of the true source rank. The bottom and top of the box show the values of the first and third quartiles. In a single realization, GMLA can have rank equal to *N* = 6299 when the score for the true source is undefined (see Fig. 1). (**c**) The mean distance error. For *ρ* > 10% GMLA has the lowest distance error. (**d**) The mean computation time of a single realization of GMLA is substantially shorter that PTVA-LI and, in contrast to PTVA-LI, is independent of the density of observers. Each point in the figures is the result of 1000 realizations. Parameters: $\lambda =\sqrt{2}$, *K*_0_ = 30 (details in the text).

Another test was performed on Gnutella, a real peer-to-peer network. This kind of network is used for direct exchange of data via Internet between users and therefore can be used to spread the malware. The graph obtained from SNAP Datasets^38–40^ contains *N* = 6299 nodes and has the average degree 〈*k*〉 = 6.6 (more details on data are in the section Methods). We examine the algorithms for different densities of the observers, but we keep a constant number of the nearest observers in GMLA (*K*_0_ = 30). During tests we use simple SI model to simulate spreading. The results are shown in Fig. 5. For the density of the observers below 10% the outcomes of both methods are very similar – GMLA has slightly better accuracy but visibly worse rank than PTVA-LI. The situation changes when the density of the observers is equal or greater than 10% – GMLA performs better according to all efficiency measures. However, the main difference between these algorithms lies in the computation time (Fig. 5b). Initially, for *ρ* = 2.5% the computation time differs by a factor 61.5, but it increases with the density of observers since the computation time for PTVA-LI increases with *ρ* (see Fig. S2d in Sup. Inf. Section S.1).

## Discussion

We introduce a new algorithm (GMLA) for the spread source localization in the well-known Pinto-Thiran-Vetterli limited observers formulation. The main drawback of the Pinto-Thiran-Vetterli Algorithm (PTVA) is its time complexity. For large networks with many observers the complexity of PTVA is defined by the complexity of matrix operations, which is *O*(*K*^3^) per node in the worst case (where *K* denotes the number of observers). We avoid this drawback in out algorithm by reducing the number of the observers used to determine the score (the likelihood of being the source) and by limiting the number of suspected nodes. The latter is performed by the selection procedure which starts from the neighbors of the first observer and follows the gradient of the score. As a result of the selection, we get a limited number of the suspected nodes ${N}_{0}=|{V}_{s}|\sim \,\mathrm{log}\,N$ in contrast to PTVA where each node is checked (*V*_*s*_ = *V*). Thanks to this approach, the complexity of Gradient Maximum Likelihood Algorithm (GMLA) is *O*(log(*N*)*N*^2^) in the worst case and as far as we know this is the fastest algorithm for the spread source detection in generic networks with incomplete observations.

**Figure 6:**
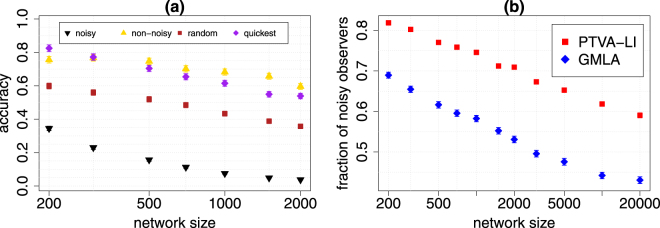
Effect of hubs in BA network (*m* = 3 and $\lambda =\sqrt{2}$ in all simulations). (**a**) Accuracy of the source localization vs network size for 4 sets of observers. Each set contains 15 observers which are 2 hops from the true source. Black triangles is the set containing only observers which are “behind” the hubs (noisy observers). Gold triangles is the set with only non-noisy observers. Dark red squares is the set with randomly selected observers. Purple diamonds is the set with the observers with the smallest time delays. These observers are the quickest receivers. Each point is the result of 2000 realizations. (**b**) The fraction of the observers which are “behind” the hubs (noisy observers) for PTVA-LI and GMLA. The analysis was done on BA network (*m* = 3) with constant density of observers *ρ* = 0.2 and the number of the nearest observers ${K}_{0}=0.5\sqrt{N}$. The results are averaged over 1000 realizations.

We test GMLA and PTVA-LI on Erdös-Rényi, Barabási-Albert and Gnutella networks and compare performance of these algorithms using three measures: the accuracy, the rank of true source, and the distance error. Both algorithms work noticeably better for ER graphs than BA networks. For ER graphs, the quality of source localization by both algorithms is similar (with a minimal advantage of PTVA-LI), but for BA networks GMLA achieves much better results. The additional tests performed on Regular Random Graphs (Fig. S19 in Sup. Inf. Section S.4), Exponential Random Graph (Figs S20, S21 in Sup. Inf. S.4) and Configuration Model with the degree distribution which follows a power-law (Fig. S22 in Sup. Inf. S.4) confirm that GMLA outperforms PTVA-LI for scale-free networks. As is well known, the essential property of scale-free network is existence of the hubs - the nodes with a very high degree (here we consider nodes with $k\geqslant \sqrt{N}$ to be the hubs). The hubs are usually responsible for a very rapid spread in the network, but can their presence hinder detection of the source? Fig. 6a shows the accuracy of PTVA-LI for 4 special sets of observers in BA network. All sets are equipotent (15 nodes) and contain only the observers which are the second order neighbors of the true source. In addition, the first set (black triangles) consists solely of the observers which are “behind” the hubs. We say the observer is “behind” the hub (or is noisy) if the shortest path between this observer and the true source passes through any hub. This also applies to the observers which are the hubs. The second set (gold triangles) is the opposite of the first set - it contains only non-noisy observers which are not “behind” any hub. The third set (dark red squares) is a random mixture of the first two. The last set (purple diamonds) consists of the observers which have the smallest times at which the spread reached them (the quickest observers). This is the same criterion for the selection of observers as that which GMLA uses. As Fig. 6a shows, using the observers “behind” the hubs substantially worsens the accuracy of PTVA-LI. It means that information is degraded after passing through the hub. This is the main reason why PTVA-LI and GMLA are less effective for scale-free networks. The highest accuracy of PTVA-LI is achieved when using only non-noisy observers. However, the quality of the source localization of the algorithm with the quickest observers is only slightly lower. Since GMLA uses the quickest observers, it achieves better results than PTVA-LI in scale-free networks with hubs, because the nearest observers infrequently are “behind” the hubs for sufficiently large networks, as is confirmed by Fig. 6b. Moreover, this conclusion is supported by the results obtained for Gnutella network, which also contains some hubs (0.4% of nodes has degree $k\geqslant \sqrt{N}$).

Although GMLA does not use information from all observers, as PTVA-LI does, it achieves better results for scale-free networks in quality of localization tests based on three measures: the accuracy, the rank of true source, and the distance error. This is because GMLA acts like a filter and rejects low quality information from distant observers which are often “behind” the hubs.

In summary, we proposed a new method for fast and accurate detection of spread source with incomplete observations which is capable to process timely large networks consisting of tens of thousands of nodes. Our algorithm is much faster and provides higher quality of localization results than Pinto-Thiran-Vetterli algorithm for scale-free networks. The key to this success is limiting the information sources to the most important observers, while ignoring excessive and noisy information from far observers, as well as use of likelihood gradient for selection of potential spread sources. The phrase “less is more” once again turned out to be truth here.

## Methods

### Propagation ratio

For spreading process we define the propagation ratio *λ* as the ratio between the mean *μ* and the standard deviation *σ* of time delay associated with an edge in the network.

### Susceptible-Infected (SI) model

We simulate the spread through the network using discrete Susceptible-Infected (SI) model^41^. In this model each node can be in one of two states: susceptible or infected. At *t* = 0 only one random node is infected. We called this node the true source. At each subsequent time step each infected node has a chance to pass the information to its neighbor. The number of chances per time step is equal to the number of neighbors and for each neighbor the probability of success *β* is the same. The parameter *β* is called the infection rate. Since the number of time steps needed to pass the information from one node to its neighbor is equal to the number of independent trials (with the probability *β*) needed for first occurrence of success, it is described by the geometric distribution and therefore the mean propagation time per edge is *μ* = 1/*β* and the variance is *σ*^2^ = (1−*β*)/*β*^2^. It follows that the propagation ratio *λ* = *μ*/*σ* for SI model is $\lambda =1/\sqrt{1-\beta }$.

### Efficiency measures

#### Accuracy

The accuracy of a single realization is ${a}_{i}=1/|{V}_{top}|$ if s^*^∈ *V*_*top*_ or *a*_*i*_ = 0 otherwise, where s^*^ is the true source and *V*_*top*_ is a group of nodes with the highest score (top scorers). The total accuracy *a* is an average of many realizations *a*_*i*_, therefore *a* ∈ [0,1]. This measure takes into account the fact that there might be more than one node with the highest score (ties are possible).

#### Rank

The rank is the position of the true source on the node list sorted in descending order by the score. In other words this measure shows how many nodes, according to an algorithm, is a better candidate for a source than the true source. If the real source has exactly the same score as some other node (or nodes), the true source is always below that node (these nodes) on the score list sorted in descending order. The rank takes into account the fact that an algorithm which is very poor in pointing out the source exactly (low accuracy) can be very good at pointing out a small group of nodes among which is the source.

#### Distance error

The distance error is the number of hops (edges) between the true source and a node designated as the source by an algorithm. If |*V*_*top*_| > 1, which means that an algorithm found more than one candidate for the source, the distance error is computed as a mean shortest path distance between the real source and the top scorers.

### Baseline method

The baseline method serves as the benchmark for accuracy and distance error tests. It assumes that the real source is the first observer reporting the spread. The baseline method works in no time and its accuracy is expected to be equal to the density of observers; this follows from the fact that if the true source is among the observers, it has to be the observer with the smallest arrival time. One can expect a quite low value of the mean distance error in this case, because the baseline method never makes big mistakes in terms of distance from the true source. Apart the poor accuracy, the baseline method does not assign the scores to the nodes which means that it cannot be used to find the rank of the real source.

### Gnutella peer-to-peer network

We used the data from SNAP Datasets^38–40^. This dataset consists of a snapshot of the Gnutella peer-to-peer file sharing network from 8 August 2002. Nodes represent hosts in the Gnutella network topology and edges represent connections which were established on 8 August 2002. The data has been anonymized by the researchers from Stanford University before it was made available. The graph contains *N*_*tot*_ = 6301 nodes and *E*_*tot*_ = 20777 edges, but we use the largest connected component which consists of *N* = 6299 nodes and *E* = 20776 edges (〈*k*〉 = 6.6). The diameter of the network is 9, the average path length is 3.7 and the average clustering coefficient is 0.0109.

### Testbed

The time tests were performed in Java 7 using AMD FX-8350 4 GHz processor. We used jblas v.1.2.4^42^ as a fast linear algebra library for Java.

## Electronic supplementary material

Supplementary information
