# Supplementary material for: Fast and accurate detection of spread source in large complex networks
Source: Sci Rep. 2018 Feb 6;8:2508. doi: 10.1038/s41598-018-20546-3 (PMC5802743; doi:10.1038/s41598-018-20546-3)
Supplement: Supplementary file 1 — Supplementary information [file 41598_2018_20546_MOESM1_ESM.pdf]

Fast and accurate detection of spread source in large complex networks

Supplementary Information

Robert Paluch[1,*], Xiaoyan Lu[2], Krzysztof Suchecki[1], Bolesław K. Szymański[2,3], and Janusz A. Hołyst[1,4]

[1]Center of Excellence for Complex Systems Research, Faculty of Physics, Warsaw University of Technology, Koszykowa 75, 00662 Warsaw, Poland

[2]Social Cognitive Networks Academic Research Center, Rensselaer Polytechnic Institute, 110 8th Street, Troy, NY, 12180-3590 USA

[3]The ENGINE Centre, Wroclaw University of Science and Technology, Wyb. Wyspianskiego 27, 50-370 Wroclaw, Poland

[4]ITMO University, 49 Kronverkskiy av., 197101 Saint Petersburg, Russia

[*]paluch@if.pw.edu.pl

January 5, 2018

S.1 Pinto-Thiran-Vetterli Algorithm

The algorithm proposed by Pinto, Thiran and Vetterli is a deterministic Gaussian method for a single source identification in a network of size N. It requires a full knowledge of the network structure $\mathcal{G}$ and of times t_k, $1 \leqslant k \leqslant K$, at which the information arrived at observer k (*observed delays*). The observers are nodes that report report from which neighbor and at what time it received the information. One observer (lets say o_K) serves as a reference point. The method also assumes the information spreads through the network along the shortest paths and the propagation times θ_i for each edge are i.i.d Gaussian random variables, for which the mean μ and the variance σ^2 are known. The assumption of diffusion along the shortest paths is used when the algorithm is applied to an arbitrary, non-tree graph. In this case the actual propagation tree (which is not known a priori) is approximated by a breadth-first search (BFS) tree composed of the shortest paths between the node which is supposed to be the source and all observers. The algorithm calculates a score for each node s using a density function of $(K-1)$-dimensional normal distribution, where K is the number of observers. The node with the highest score is pointed as the rumor source. If $|\mathcal{P}(u,v)|$ denotes the length of the path $\mathcal{P}(u,v)$ connecting nodes u and v, $\mathbf{d}$ is the vector of observed delays, $\boldsymbol{\mu}_s$ is the vector of deterministic delays and $\mathbf{\Lambda}_s$ is the delay covariance matrix, the score of node s is given by:

$$\phi(s) = \frac{\exp(-\frac{1}{2}(\mathbf{d}-\boldsymbol{\mu}_s)^{\mathrm{T}}\mathbf{\Lambda}_s^{-1}(\mathbf{d}-\boldsymbol{\mu}_s))}{|\mathbf{\Lambda}_s|^{1/2}}, \tag{1}$$

where

$$[\mathbf{d}]_k = t_k - t_K, \tag{2}$$

$$[\boldsymbol{\mu}_s]_k = \mu(|\mathcal{P}(s,o_k)| - |\mathcal{P}(s,o_K)|), \tag{3}$$

$$[\mathbf{\Lambda}_s]_{k,i} = \sigma^2|\mathcal{P}(o_K,o_k) \cap \mathcal{P}(o_K,o_i)|, \tag{4}$$

for $k, i = 1, \dots, K-1$. Despite the simplifications, the method works reasonably well under certain conditions (density of observers $\rho = K/N > 10\%$ and propagation ratio $\lambda = \mu/\sigma > \sqrt{2}$).

PTVA uses the whole network for inferring the source, which makes the algorithm computationally expensive. It calculates the score for each node in the network which requires to create BFS tree N times. Each BFS tree covers practically the entire network since it reaches to all observers, including these which are far from the root node. The complexity of creating BFS tree is $O(N^2)$ per node $s \in \mathcal{G}$. Unfortunately, the assumption of diffusion along the shortest paths works well only for the observers which are close to the suspected node (the root of BFS tree) since it makes use of the fact that typical random networks are locally tree-like. However, this may not be true for observers which are far from the supposed source. Thus making use of the distant observers may be problematic – it increases the expenses and decreases the correctness (because there can be many possible paths between the source and the remote observers). So far we have considered the situation when the number of observers is much smaller than the number of other nodes. In this case the overall complexity of PTVA is $O(N)$ for arbitrary trees and $O(N^3)$ for general graphs. But what if the number of the observers is not constant and small? In order to keep quality of the results of PTVA constant while increasing the size of the network, the number of observers also should increase. Assuming constant density of observers $\rho = K/N$, the complexity increases to $O(N^2)$ for arbitrary trees and $O(N^4)$ for general graphs (in the worst case), which makes the algorithm difficult to apply to large networks. As numerical results show (Fig. S1), when K is increasing, matrix operations in Eq. 1 (especially inverting and obtaining determinant) are more computationally demanding than creating BFS tree since they have time complexity $O(K^3)$ in the worst case. In general, the algorithm's complexity for arbitrary graphs as $O(N(K^3 + N^2))$. Assuming $K \sim N^\gamma$, PTVA complexity ranges from $O(N^3)$ when $\gamma \leqslant 2/3$ to $O(N^4)$ when $\gamma = 1$.

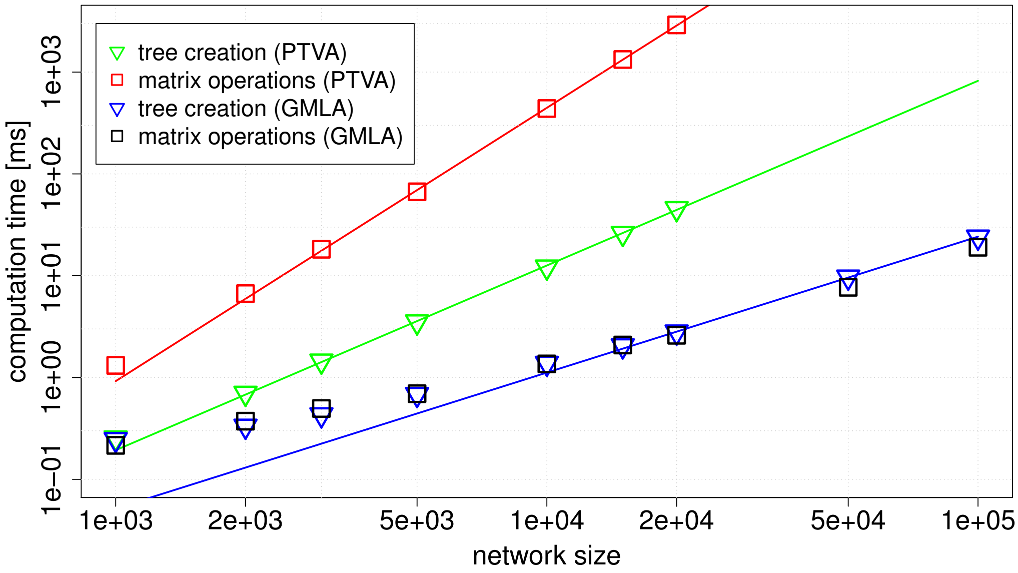

Figure S1: The comparison of the times needed for creation of the propagation tree and for matrix operations. The time complexity of matrix operation for PTVA is $O(N^{2.68})$ per node (red line), while the time complexity of tree creation is $O(N^{1.81})$ per node for PTVA (green) and $O(N^{1.34})$ for GMLA (blue). The tests were performed on BA network with $m = 3$. The observers were distributed randomly over whole network with density $\rho = 0.1$.

The results of selected studies on the localization quality of Pinto-Thiran-Vetterli Algorithm executed on data with Limited Information (PTVA-LI) are presented in figures S2 and S3. The explanation and motivation of PTVA-LI can be found in Section Pinto-Thiran-Vetterli Algorithm in the main paper. Figure S2 shows how the algorithm's quality of localization depends on the density of observers. We present these results for three different values of infection rates: $\beta \in \{0.2, 0.5, 0.8\}$. As seen in Fig. S2, increasing the density of observers improves the quality of the results to some extent. The higher the infection rate is, the faster the accuracy curve saturates on the level which is equal to the infection rate. This means that the accuracy of PTVA-LI does not converge to unity for $\rho \to 1$ if $\beta < 1$. On the other hand the computation time increases very fast with the density of observers, i.e. $t \sim \rho^2$. Figure S3 presents dependency of the localization quality on the infection rate for three different values of the density of observers $\rho \in \{0.1, 0.2, 0.3\}$. One can see that the infection rate is a crucial factor for the quality of results of PTVA-LI. For $\beta < 0.5$ (which corresponds to propagation ratio $\lambda < \sqrt{2}$) PTVA-LI has worse accuracy and distance error than the baseline method. For $0.5 \leqslant \beta < 0.6$ PTVA-LI has better accuracy than the baseline method but still worse distance error. For $\beta \geqslant 0.5$ PTVA-LI finally overtakes the baseline method. Our studies demonstrate that PTVA-LI works best with small density of observers $\rho \approx 0.2$ (due to the short calculation time) and high propagation ratio (the higher λ, the better performance).

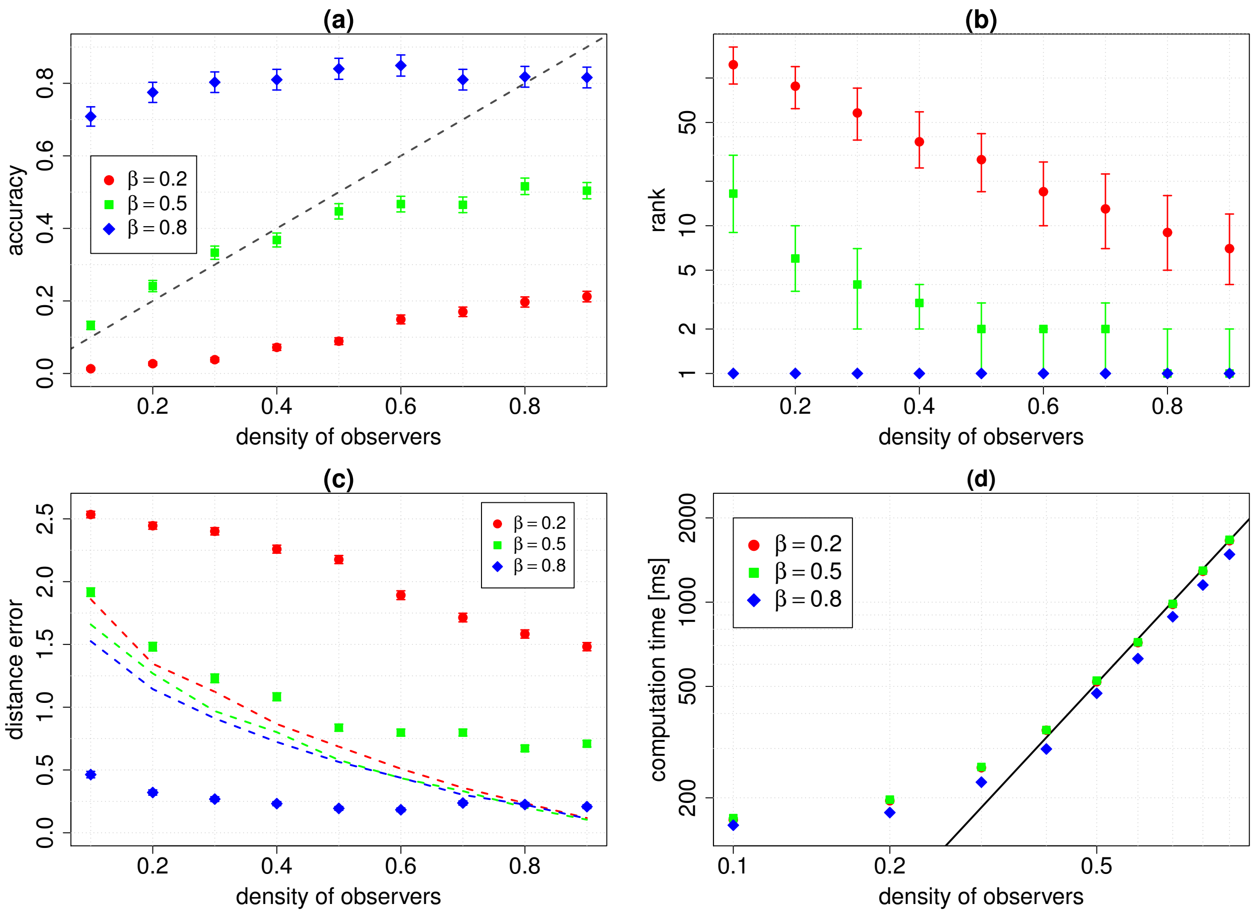

Figure S2: Performance of PTVA-LI versus the density of observers ρ. The tests were performed on BA network with $N = 500$ and $m = 3$. SI model was used for signal propagation with infection rates $\beta = \{0.2, 0.5, 0.8\}$ which gives values of propagation ratio $\lambda = \{\sqrt{5}/2, \sqrt{2}, \sqrt{5}\}$. Each point is the result of 1000 realizations. (a) Accuracy. The dotted line shows the performance of the baseline method which is equal to the density of observers. (b) Median rank of the true source. The error bars indicate a spread between 4th and 6th decile. (c) Mean distance error. Dotted lines show performance of the baseline method. Colors of the lines correspond to various infection rates. (d) Mean computation time. The solid line is a power law with the exponent $\alpha = 2$.

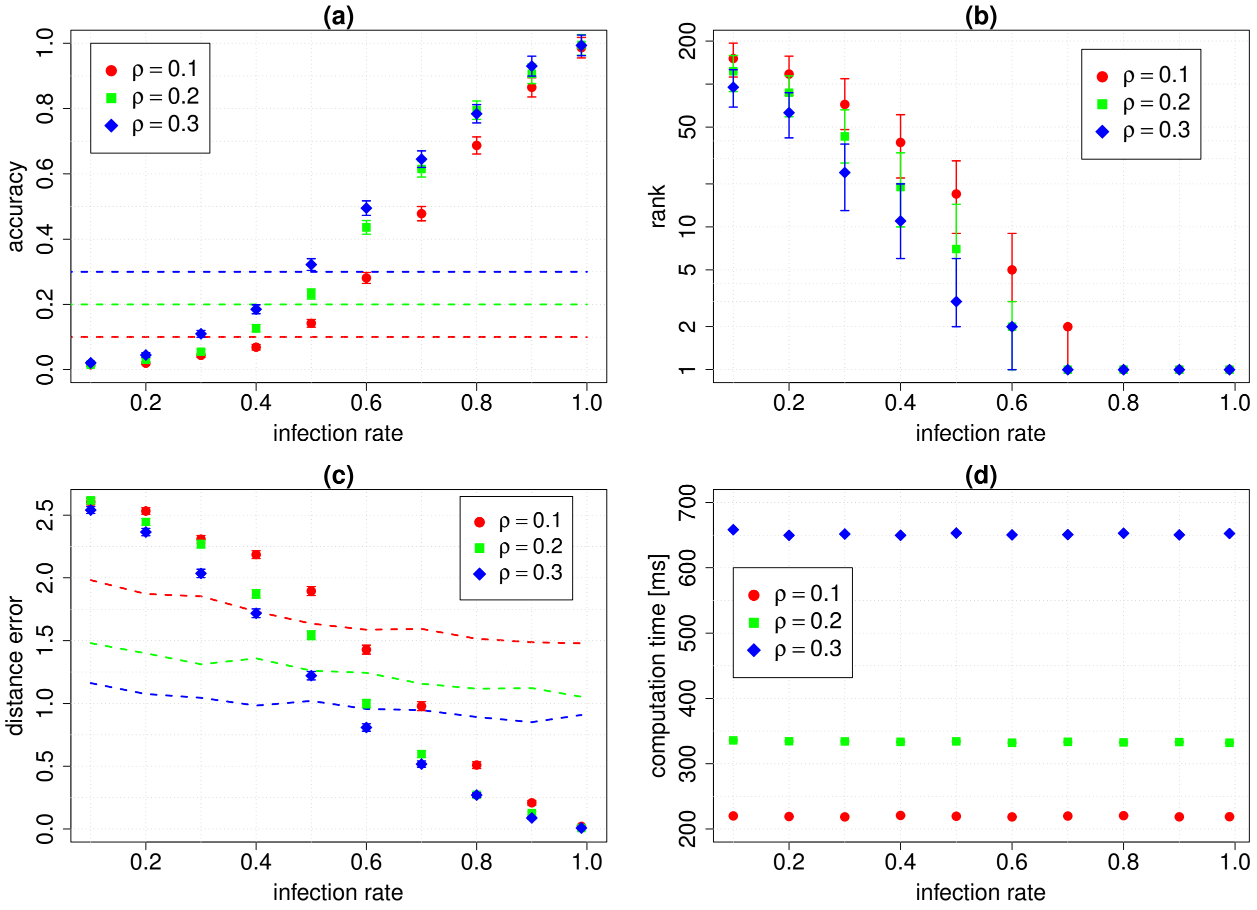

Figure S3: Performance of PTVA-LI versus the infection rate β. The tests were performed on BA network with $N = 500$ and $m = 3$. The observers were distributed randomly over whole network with densities $\rho = \{0.1, 0.2, 0.3\}$. Each point is the result of 1000 realizations. (a) Accuracy. The dotted lines show performance of the baseline method which is equal to density of observers. (b) Median rank of the true source. The error bars indicate a spread between 4th and 6th decile. (c) Mean distance error. The dotted lines show performance of the baseline method. The colors of the lines correspond to the density of observers. (d) Mean computation time.

S.2 Gradient Maximum Likelihood Algorithm

GMLA always uses K_0 fastest informed observers (which we call "the nearest" for simplicity) and skips the others. Thus, the question arises if the observers which receive the signal later do not provide valuable additional information. Figure S4 shows how the score changes if the algorithm takes into account increasing number of the observers sorted in ascending order of the time of receiving the signal. It also shows that the first dozen or so nearest observers provide enough information to estimate the score of a node. Taking more of them does not substantially affect the result. The question remains whether the nearest observers are the best choice. Perhaps taking into account only the latest observers will provide equally good or better results? Figure S5 presents the tests performed using various groups of observers. The total number of observers is 200 and they are partitioned into 10 non-overlapping groups. The first group contains first 20 observers with the lowest delays. The second group consists of the observers from positions 21-40. The last, tenth group contains 20 observers which were informed at the latest. These results show that only the nearest observers provide sufficiently accurate information to locate the true source.

The basis of GMLA are two fundamental ideas: smartly limiting the number of observers and a gradient-like selection of suspected nodes. We have studied the effect of each of them on performance of the algorithm. The basic premise behind the selection of suspected nodes is saving time spent on calculating the scores. The number of suspected (and checked) nodes in GMLA is proportional to the logarithm of the network size (Fig. S6a), and therefore much smaller than the number of suspected nodes in PTVA-LI. Figure S7 shows the quality of the localization results of the algorithm with the gradient-like selection. These results show that the gradient-like selection not only decreases the time of computation (Fig. S7d) but also improves the accuracy, the rank of true source and the distance error. Table S1 explains why the gradient-like selection improves quality of the source detection. The first column shows that PTVA-LI always finds the true source if the score of the source is the global maximum (23.9% of all cases). Otherwise, PTVA-LI is always wrong (76.1% of all cases). In comparison, GMLA (but without limiting the number of observers) sometimes miss the true source even though the score of the source is the best score in the network (1.5% of all cases), but in some cases it can detect the source which has not the maximum score (5.8% of all cases). Figure S8 shows the quality of the localization results of the algorithm which uses the limited number of the nearest observers, but does not use the gradient-like selection of suspected nodes. The accuracy of such an algorithm increases with the network size and outperforms PTVA-LI for networks of size $N = 500$ or bigger. However, this is true only for scale-free networks. As we write in Discussion in the main paper, performance improvement by limiting the number of observers is due to rejecting observers which are "behind the hubs". The substantial computation time reduction by limiting the number of observers is due to a big acceleration of matrix operations (see Fig. S1).

Table S1: The results of 1000 realizations of PTVA-LI/GMLA on BA network ($N = 1000$, $\langle k \rangle = 6$). The numbers on the left side of the cells refer to PTVA-LI. The symbol $\phi(s^*)$ denotes the score of the true source, while $\max(\phi)$ is the highest score in the network in a given realization.

	$\phi(s^*) = \max(\phi)$	$\phi(s^*) < \max(\phi)$
# hits	239 / 224	0 / 58
# mishits	0 / 15	761 / 703

Figures S9 and S10 present the results of the performance tests of GMLA for BA network. A very substantial difference between PTVA and GMLA is that GMLA computes and assigns scores to limited number of nodes and therefore sometimes it can skip the real source. When this happens, the node which is the true source is left without the score thus its rank is equal to the size of the network (it occupies the last place on the score ranking ex aequo with the other skipped nodes). Figures S9a and S10a show how often GMLA assigns the score to the node which is the true source. The probability $P(s^* \in V_s)$ is a monotonically increasing function of ρ and β which is 0.85 for $\rho = 0.2$ and $\beta = 0.5$ (which are typical values used in our studies). The fact that $P(s^* \notin V_s) < 1$ weakens the performance of GMLA but our studies show that this is a case only when ρ or β are

very small. On the other hand, when the propagation ratio is very high (β close to 1) PTVA-LI performs better than GMLA. However, in real life the propagation ratio is rather low, because the spreading process is highly nondeterministic. In cases of low and medium β, GMLA performance dominates over PTVA-LI performance despite the fact that GMLA sometimes rejects the true source at the selection stage.

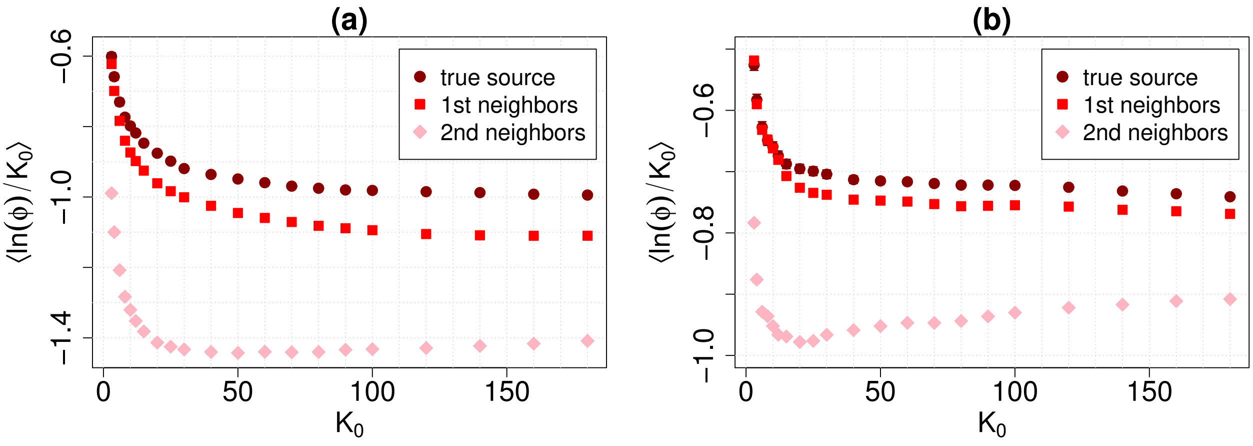

Figure S4: Normalized logarithm of the score vs the number of nearest observers which were used for the computation. The score of a node (the true source or its first neighbor or its second-order neighbor) was computed for various values of the number of the nearest observers. The results show that increasing the number of the nearest observers above a certain threshold does not change the score substantially. The tests were performed on (a) ER graph with $\langle k \rangle = 6$, (b) BA network with $m = 3$. The size of networks is $N = 1000$ The observers were distributed randomly over whole network with density $\rho = 0.2$. SI model was used for propagation with infection rates $\beta = 0.5$. The results are averaged over 100 realizations. The standard errors are smaller than symbols on plot.

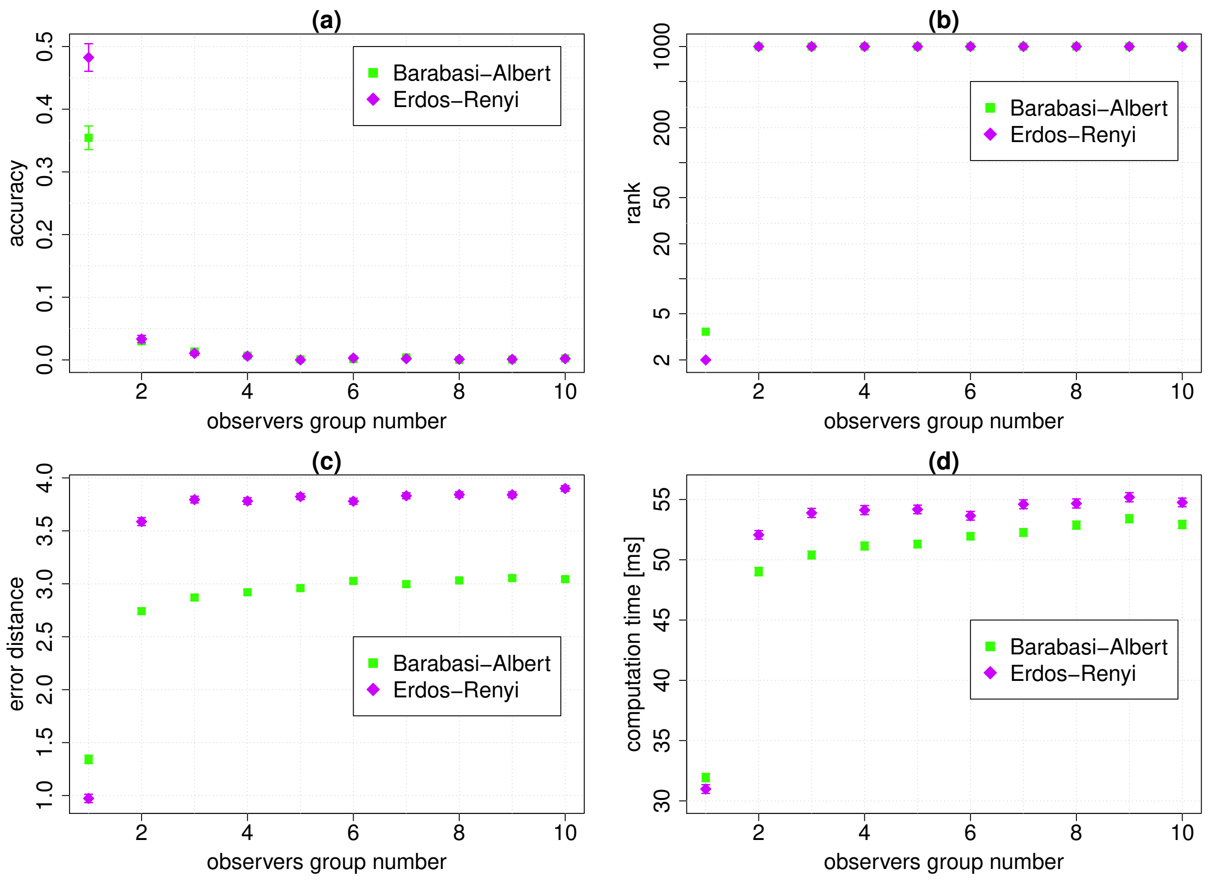

Figure S5: Performance of GMLA depending on the observations used. The observers were grouped according to the time of receipt of the information. The first group contains first 20 observers with the lowest delays. The tests were performed on BA and ER networks. (a) Accuracy drops rapidly at the transition from the first to second group. (b) Median rank of the true source. (c) Mean distance error. (d) Mean computation time. Parameters: $\langle k \rangle = 6$, $\lambda = \sqrt{2}$, $\rho = 0.2$.

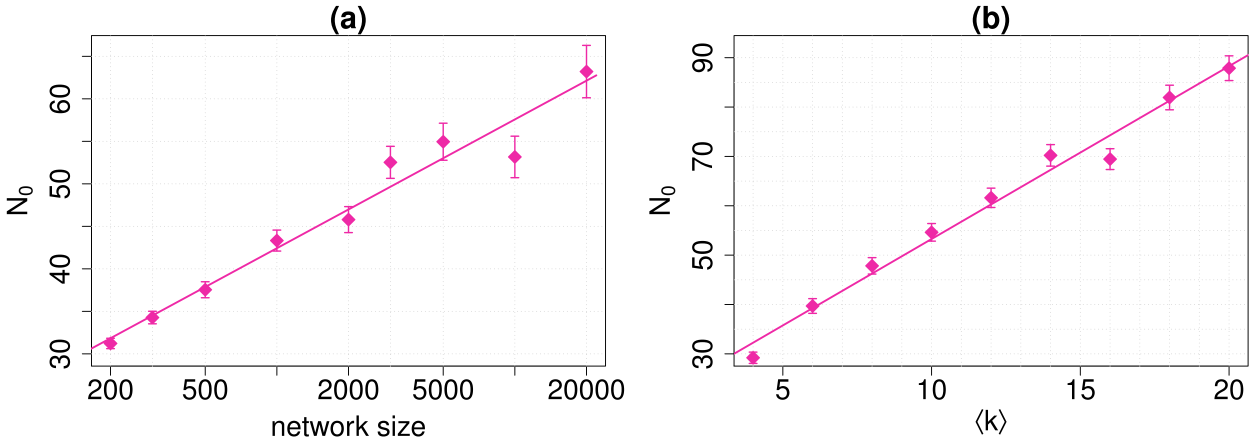

Figure S6: (a) The number of suspected nodes vs the size of BA network. The fit is a logarithmic function $N_0 = 6.58 \ln(N) - 3$. Parameters: $\langle k \rangle = 6$, $\rho = 0.2$, $\beta = 0.5$, $K_0 = 0.5\sqrt{N}$, 1000 realizations. (b) The number of suspected nodes vs the average degree of BA network. The fit is a linear function $N_0 = 3.5\langle k \rangle + 18.2$. Parameters: $N = 2000$, $\rho = 0.2$, $\beta = 0.5$, $K_0 = 20$, 1000 realizations.

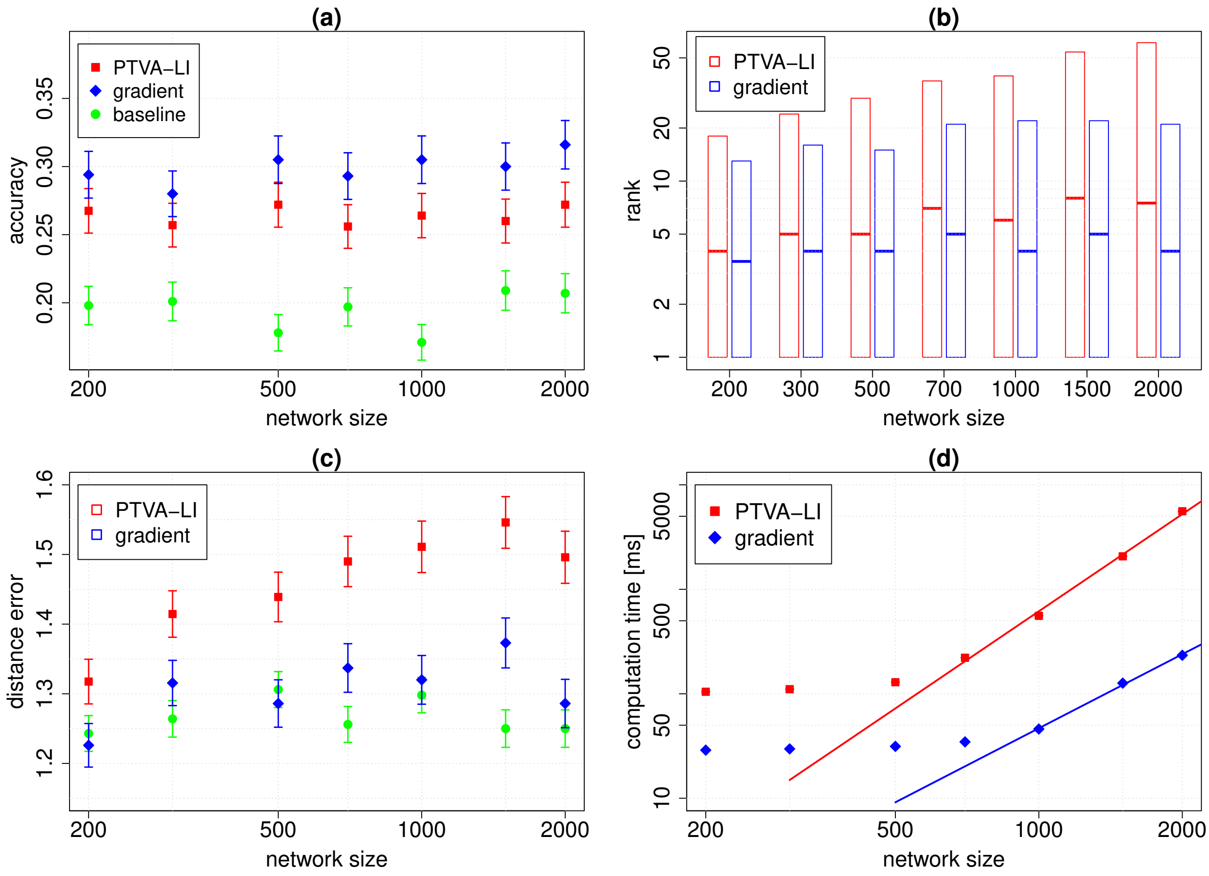

Figure S7: The result of a gradient-like selection of suspected nodes in BA network ($m = 3$). Blue diamonds are the results of the source detection obtained by applying GMLA with $K_0 = K$, i.e. without the limited number of the observers. Green dots are the results of the baseline method, which always selects the first observer as the true source. (a) Accuracy of GMLA is higher by several percent points than accuracy of PTVA-LI. (b) The band inside the box shows the median of the true source rank. The bottom and top of the box show the values of the first and third quartiles. GMLA has noticeably lower rank than PTVA-LI. (c) The mean distance error of GMLA is lower than PTVA and similar to the mean distance error of the baseline method. (d) The mean computation time of a single realization of GMLA is substantially smaller that PTVA-LI. The solid lines are power laws with exponents 3.1 (red) and 2.4 (blue). Parameters: $\langle k \rangle = 6$, $\lambda = \sqrt{2}$, $\rho = 0.2$, $K_0 = K$.

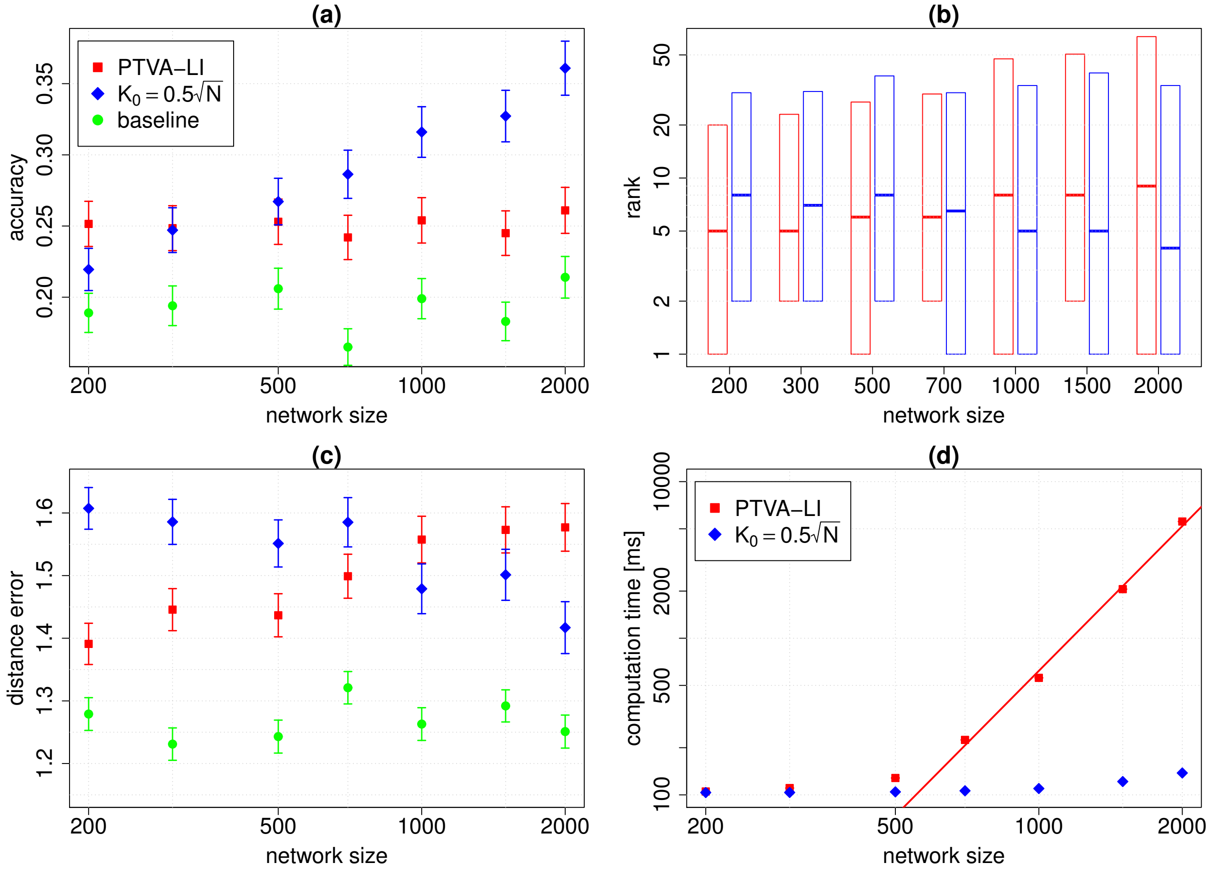

Figure S8: The result of a limited number of observers in BA network ($m = 3$). Blue diamonds are the results obtained by selecting only $K_0 = 0.5\sqrt{N}$ nearest observers (without a gradient-like selection of suspected nodes). Green dots are the results of the baseline method, which always selects the first observer as the true source. (a) Accuracy of GMLA increases with the network size and exceeds the accuracy of PTVA-LI in cases of network with 500 nodes or more. (b) The band inside the box shows the median of the true source rank. The bottom and top of the box show the values of the first and third quartiles. GMLA has lower rank than PTVA-LI for networks of size 1000 or bigger. (c) The mean distance error for PTVA-LI increases with the network size in contrast to GMLA, for which the mean distance error became smaller than PTVA-LI for networks of size 1000 or bigger. (d) The mean computation time of a single realization of GMLA is substantially smaller that PTVA-LI. The solid line is power law with exponents 3.1 (red). Parameters: $\langle k \rangle = 6$, $\lambda = \sqrt{2}$, $\rho = 0.2$, $K_0 = 0.5\sqrt{N}$.

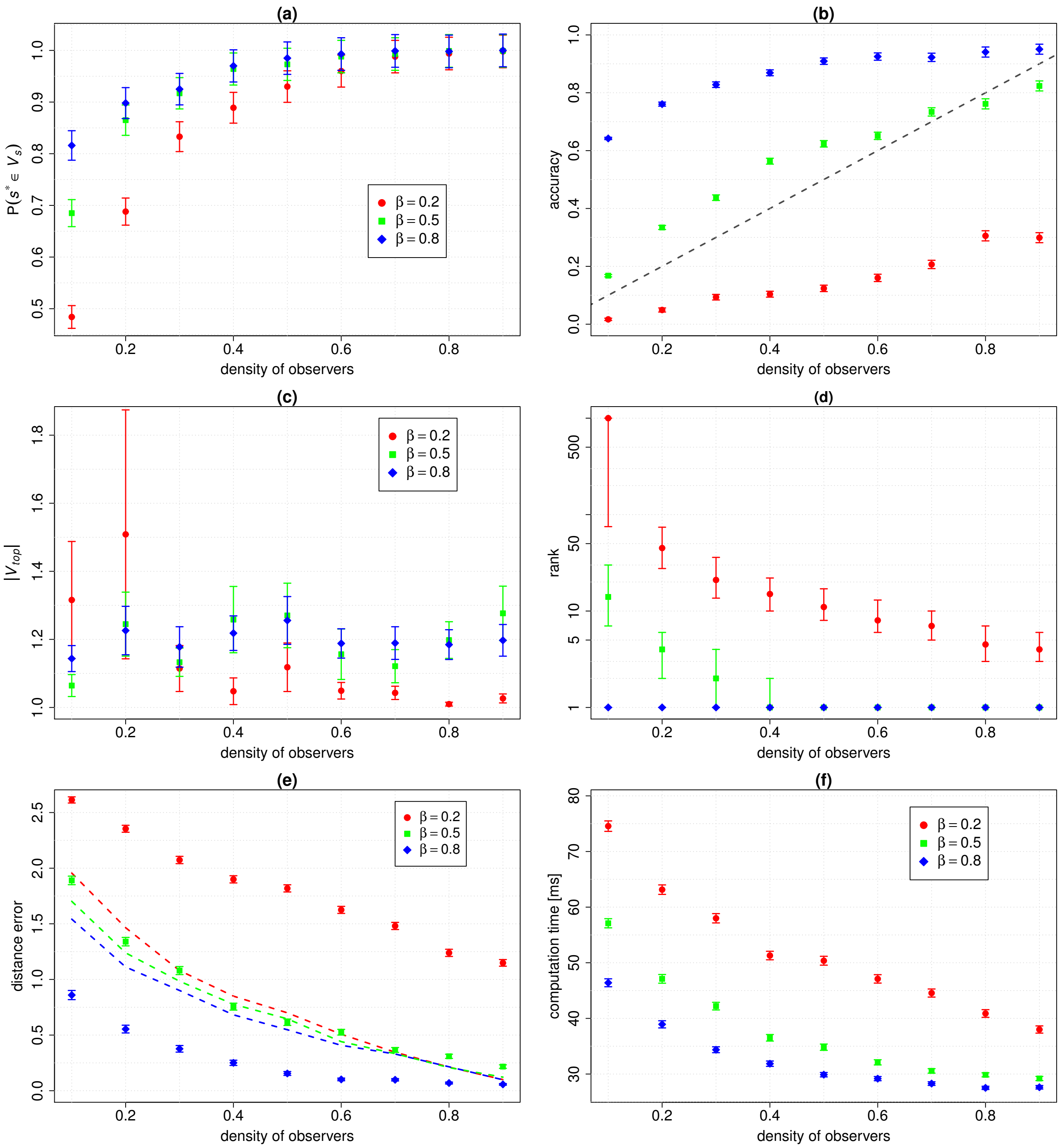

Figure S9: Performance of GMLA versus the density of observers ρ. The tests were performed on BA network with $N = 1000$ and $m = 3$. The number of the nearest observers was set to $K_0 = 30$. SI model was used for propagation with infection rates $\beta = \{0.2, 0.5, 0.8\}$ which correspond to values of propagation ratio $\lambda = \{\sqrt{5}/2, \sqrt{2}, \sqrt{5}\}$. Each point is result of 1000 realizations. (a) Empirical probability that the true source is in the group of suspected nodes (and has a calculated score). (b) Accuracy. Dotted line shows performance of the baseline method which is equal to density of observers. (c) Mean size of the group of top scorers. (d) Median rank of the true source. The error bars indicate a spread between 4th and 6th decile. (e) Mean distance error. The dotted lines show performance of the baseline method. The colors of the lines correspond to the infection rate. (f) Mean computation time.

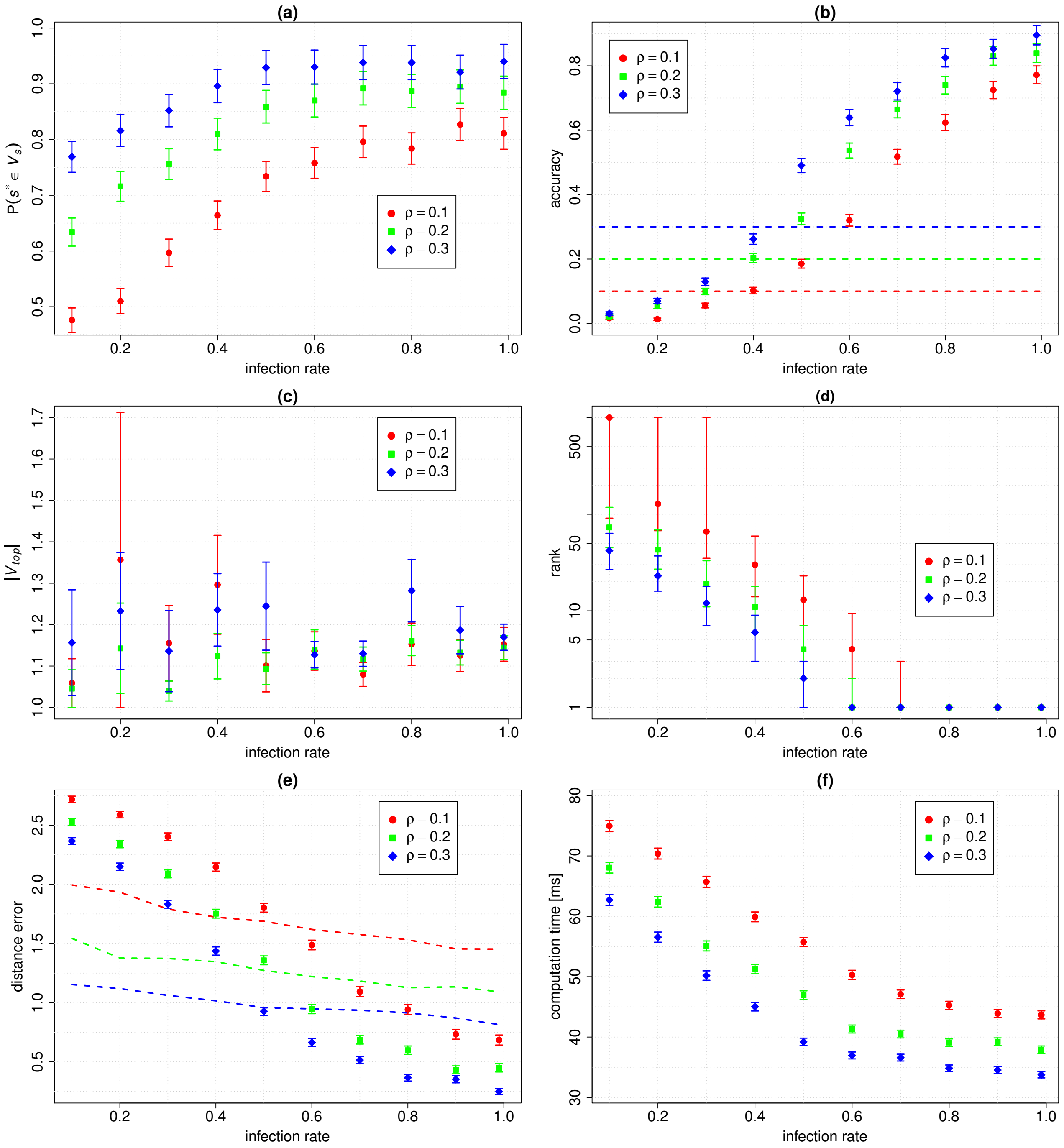

Figure S10: Performance of GMLA versus the infection rate β. The tests were performed on BA network with $N = 1000$ and $m = 3$. The number of the nearest observers was set to $K_0 = 30$. The observers were distributed randomly over whole network with densities $\rho = \{0.1, 0.2, 0.3\}$. Each point is the result of 1000 realizations. (a) Empirical probability that the true source is in the group of suspected nodes (and has a calculated score). (b) Accuracy. The dotted lines show performance of the baseline method which is equal to density of observers. (c) Mean size of the group of top scorers. (d) Median rank of the true source. The error bars indicate a spread between 4th and 6th decile. (e) Mean distance error. Dotted lines show performance of the baseline method. Colors of the lines correspond to various infection rates. (f) Mean computation time.

S.3 Optimal number of the nearest observers for GMLA

GMLA requires optimization of the one crucial parameter, namely the number of selected nearest observers K_0. This parameter controls both the precision and the speed of the algorithm. Figure S11 shows non-trivial dependency between K_0 and performance of GMLA for various sizes of BA network. Three types of behavior caused by controlling K_0 are visible. The first is seen in Fig. S11a and S11b. The probability that the true source is among the nodes with the highest score (Fig. S11a) decreases when K_0 increases. This is due to the fact, that the average size of the group of top scorers (Fig. S11b) also decreases with K_0. This is because the more observers we use, the better resolution in the score we have. The second type of dependency on K_0 can be seen in Fig. S11c,d,e,f and g. These are: the accuracy, the rank of the true source, the distance error, the number of suspected nodes, and the computation time. For each of those the optimal value of the nearest observers K_0^* exists. However, this optimal value can be different for different measures of the performance and moreover it is dependent on the size of the network (see Fig. 2d in the main article). The interesting fact is that too small value of K_0 increases the calculation time (Fig. S11f). This can be easily understood when we notice that the number of suspected nodes (Fig. S11g) also increases with small K_0. It follows that when K_0 is too small, GMLA needs more steps before it reaches the local maximum of score and therefore it computes scores for more nodes (which takes time). The last type of behavior is a linear dependency between the size of the propagation tree and the number of the nearest observers (see Fig. S11h). Regardless of the network size, the tree size is about 1.4 times larger than K_0. It is also worth mentioning that the effect of K_0 on probability of including the true source by GMLA while computing scores is rather small (Fig. S11i). Another method to find the optimal number of the nearest observers is presented in Fig. S12. We test performance of GMLA versus the size of BA network for various types of dependency between K_0 and N. The best results in terms of accuracy, rank and distance error were obtained using function $K_0 = 0.5\sqrt{N}$, which is in agreement with Fig. 2d in the main article.

We conducted the same study for Erdős-Rényi (ER) network (Fig. S13-S14). Figures S13c,d,e show that for ER graph K_0^* is a saturation point rather than a peak. However, the minimum of the computation time is still visible in Fig. S13f. In this case the optimal number of the nearest observers is the minimal number K_0 with the algorithm achieves the maximal performance. Figure S14 presents performance of GMLA versus the size of ER network for various types of dependency between K_0 and N. Again, the best results were obtained for $K_0 = 0.5\sqrt{N}$, as in the case of Barabási-Albert network.

We checked also if other factors can affect the optimal number of the nearest observers. Figures S15-S16 present dependency between K_0 and performance of GMLA for BA and ER networks of various average degrees (between 4 and 10). Despite the fact that Fig. S15c,e and S16c,e may suggest some influence of the average degree on K_0^*, it is too small to be a significance. Moreover, Fig. S15a,b,d,f,g and S16a,b,d,f,g do not confirm existence of such influence. The results of the simulations with various infection rates β (Fig. S17-S18) show that the optimal number of the nearest observers does not depend on the propagation ratio.

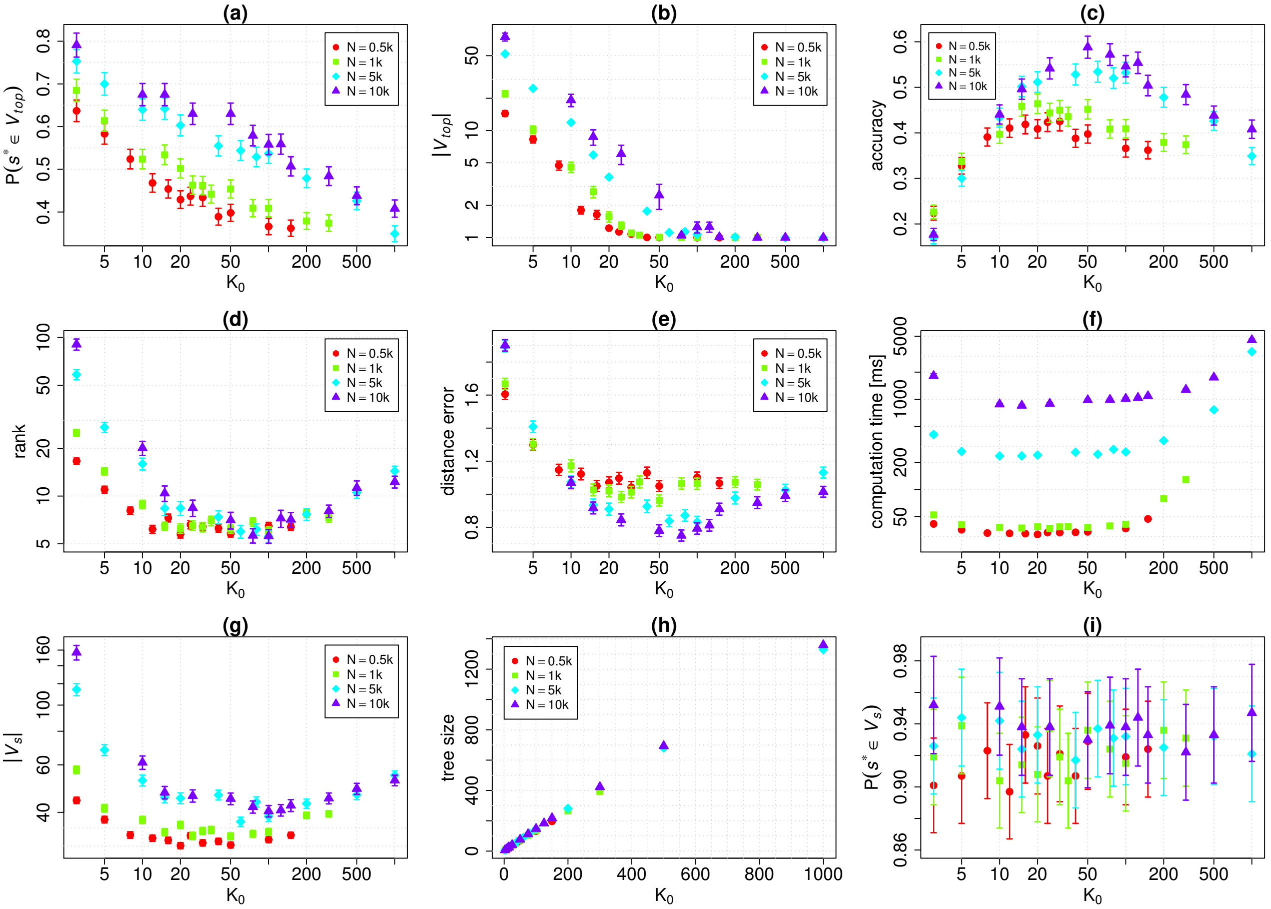

Figure S11: Performance of GMLA versus the number of the nearest observers K_0 for BA network with $m = 3$ and $N \in \{500, 1000, 5000, 10000\}$. The observers were distributed randomly over whole network with density $\rho = 0.3$. SI model was used for propagation with infection rate $\beta = 0.5$ which gives propagation ratio $\lambda = \sqrt{2}$. Each point is the result of 5000 realizations. (a) Detection rate. The empirical probability that the true source is in a group of top scorers. (b) Mean size of the group of top scorers. (c) Accuracy. (d) Mean rank of the true source. It includes only the cases when $s^* \in V_s$. (e) Mean distance error. (f) Computation time. (g) Mean number of suspected nodes $N_0 = |V_s|$ for which scores were determined. (h) Mean size of the propagation tree created for each suspected node. (i) The empirical probability that the true source is in a group of suspected nodes (and has calculated score).

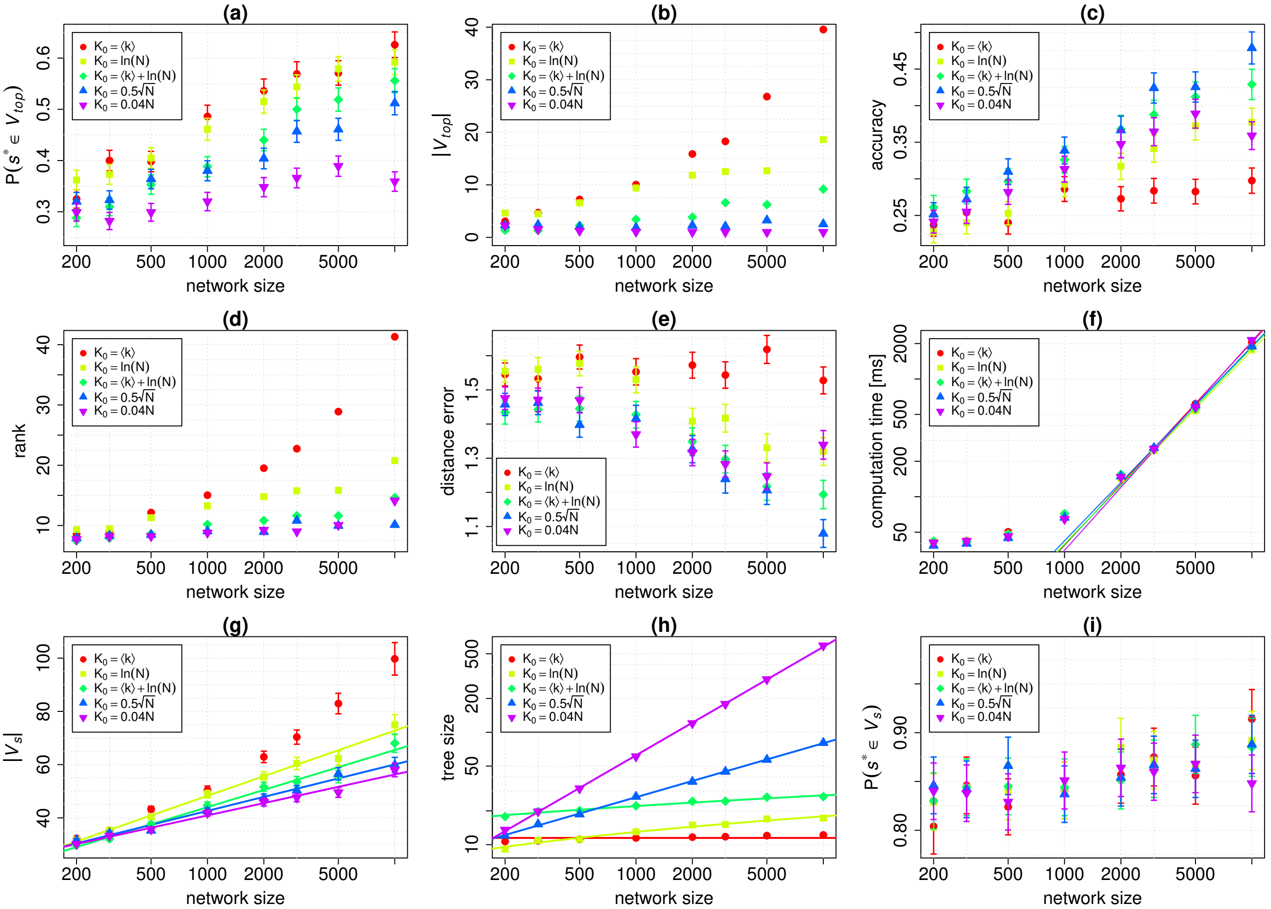

Figure S12: Performance of GMLA versus the network size N for different numbers of the nearest observers K_0. The tests were performed on BA network with $m = 3$. The observers were distributed randomly over whole network with density $\rho = 0.2$. SI model was used for propagation with infection rate $\beta = 0.5$ which gives propagation ratio $\lambda = \sqrt{2}$. Each point is the result of 1000 realizations. (a) Detection rate. The empirical probability that the true source is in a group of top scorers. (b) Mean size of the group of top scorers. (c) Accuracy. (d) Mean rank of the true source. It includes only the cases when $s^* \in V_s$. (e) Mean distance error. (f) Computation time. The solid lines are power laws with exponents: 1.64 (yellow), 1.65 (blue), 1.67 (green), 1.72 (red) and 1.77 (violet). (g) Mean number of suspected nodes $N_0 = |V_s|$ for which the scores were determined. The solid lines are linear functions of the logarithm of the network size. (h) Mean size of the propagation tree created for each suspected node. The red line is an average of the red points. The yellow and green lines are logarithmic functions. The blue and violet lines correspond to a power law with exponents 0.48 and 0.97 respectively. (i) The empirical probability that the true source is in a group of suspected nodes (and has calculated score).

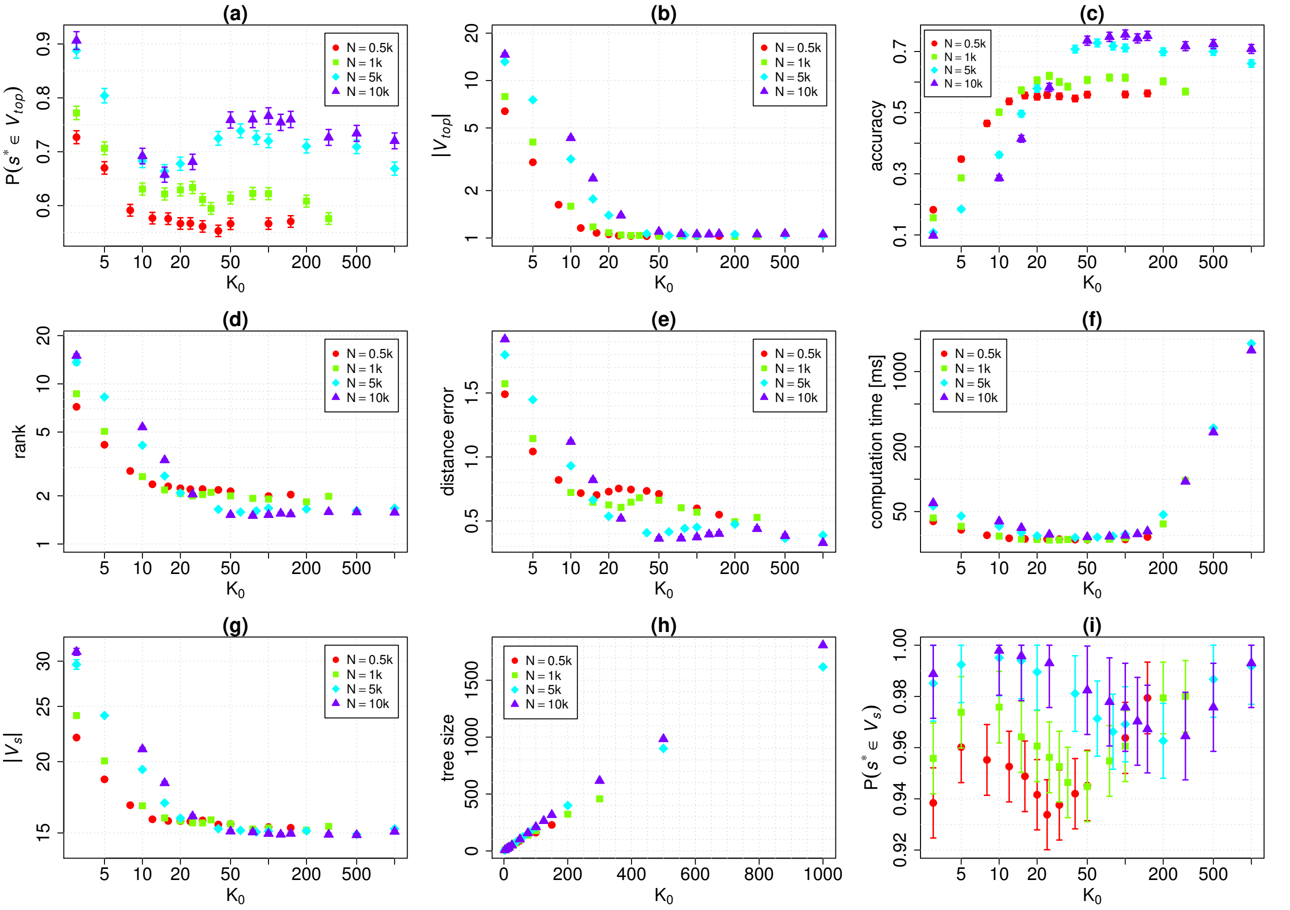

Figure S13: Performance of GMLA versus the number of the nearest observers K_0 for ER graph with $\langle k \rangle = 6$ and $N \in \{500, 1000, 5000, 10000\}$. The observers were distributed randomly over whole network with density $\rho = 0.3$. SI model was used for propagation with infection rate $\beta = 0.5$ which gives propagation ratio $\lambda = \sqrt{2}$. Each point is the result of 5000 realizations. (a) Detection rate. The empirical probability that the true source is in a group of top scorers. (b) Mean size of the group of top scorers. (c) Accuracy. (d) Mean rank of the true source. It includes only the cases when $s^* \in V_s$. (e) Mean distance error. (f) Computation time. (g) Mean number of suspected nodes $N_0 = |V_s|$ for which scores were determined. (h) Mean size of the propagation tree created for each suspected node. (i) The empirical probability that the true source is in a group of suspected nodes (and has calculated score).

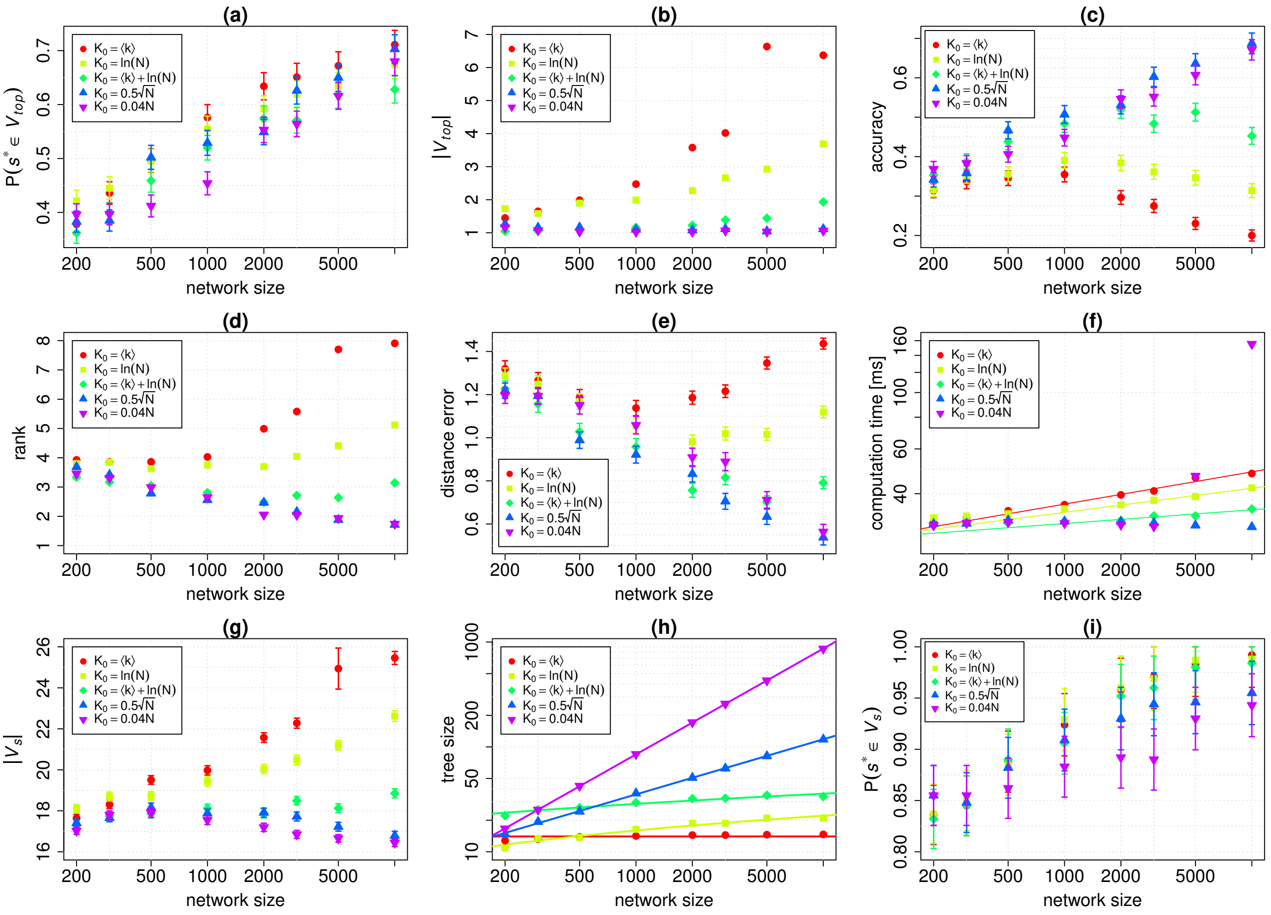

Figure S14: Performance of GMLA versus the network size N for different number of the nearest observers K_0. The tests were performed on ER graph with $\langle k \rangle = 6$. The observers were distributed randomly over whole network with density $\rho = 0.2$. SI model was used for propagation with infection rate $\beta = 0.5$ which gives propagation ratio $\lambda = \sqrt{2}$. Each point is the result of 1000 realizations. (a) Detection rate. The empirical probability that the true source is in a group of top scorers. (b) Mean size of the group of top scorers. (c) Accuracy. (d) Mean rank of the true source. It includes only the cases when $s^* \in V_s$. (e) Mean distance error. (f) Computation time. The solid lines are power laws with exponents: 1.64 (yellow), 1.65 (blue), 1.67 (green), 1.72 (red) and 1.77 (violet). (g) Mean number of suspected nodes $N_0 = |V_s|$ for which the scores were determined. The solid lines are linear functions of the logarithm of the network size. (h) Mean size of the propagation tree created for each suspected node. The red line is an average of the red points. The yellow and green lines are logarithmic functions. The blue and violet lines correspond to a power law with exponents 0.48 and 0.97 respectively. (i) The empirical probability that the true source is in a group of suspected nodes (and has calculated score).

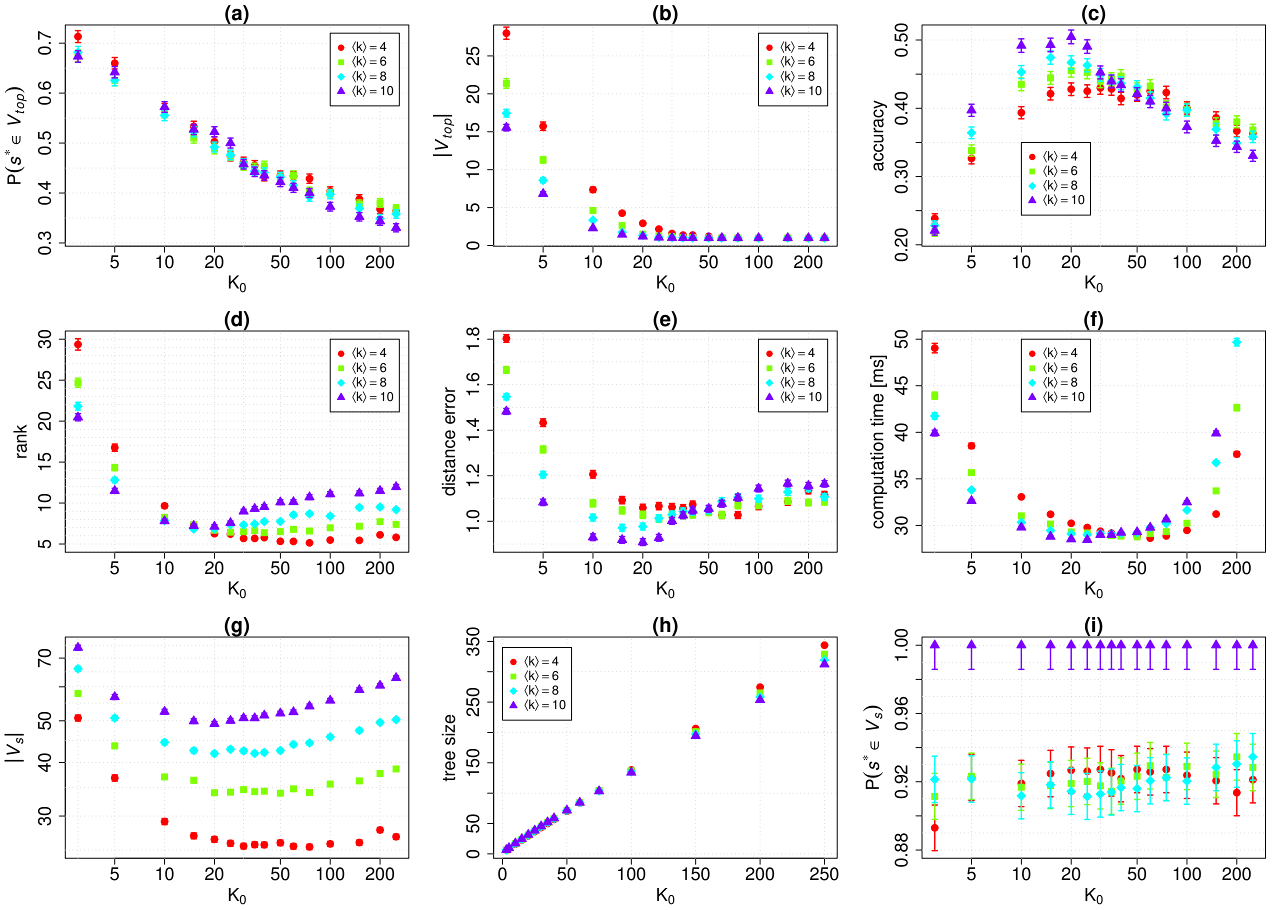

Figure S15: Performance of GMLA versus the number of the nearest observers K_0 for BA network with $N = 1000$ and $m \in \{2, 3, 4, 5\}$. The observers were distributed randomly over whole network with density $\rho = 0.3$. SI model was used for propagation with infection rate $\beta = 0.5$ which gives propagation ratio $\lambda = \sqrt{2}$. Each point is the result of 5000 realizations. (a) Detection rate. The empirical probability that the true source is in a group of top scorers. (b) Mean size of the group of top scorers. (c) Accuracy. (d) Mean rank of the true source. It includes only the cases when $s^* \in V_s$. (e) Mean distance error. (f) Computation time. (g) Mean number of suspected nodes $N_0 = |V_s|$ for which scores were determined. (h) Mean size of the propagation tree created for each suspected node. (i) The empirical probability that the true source is in a group of suspected nodes (and has calculated score).

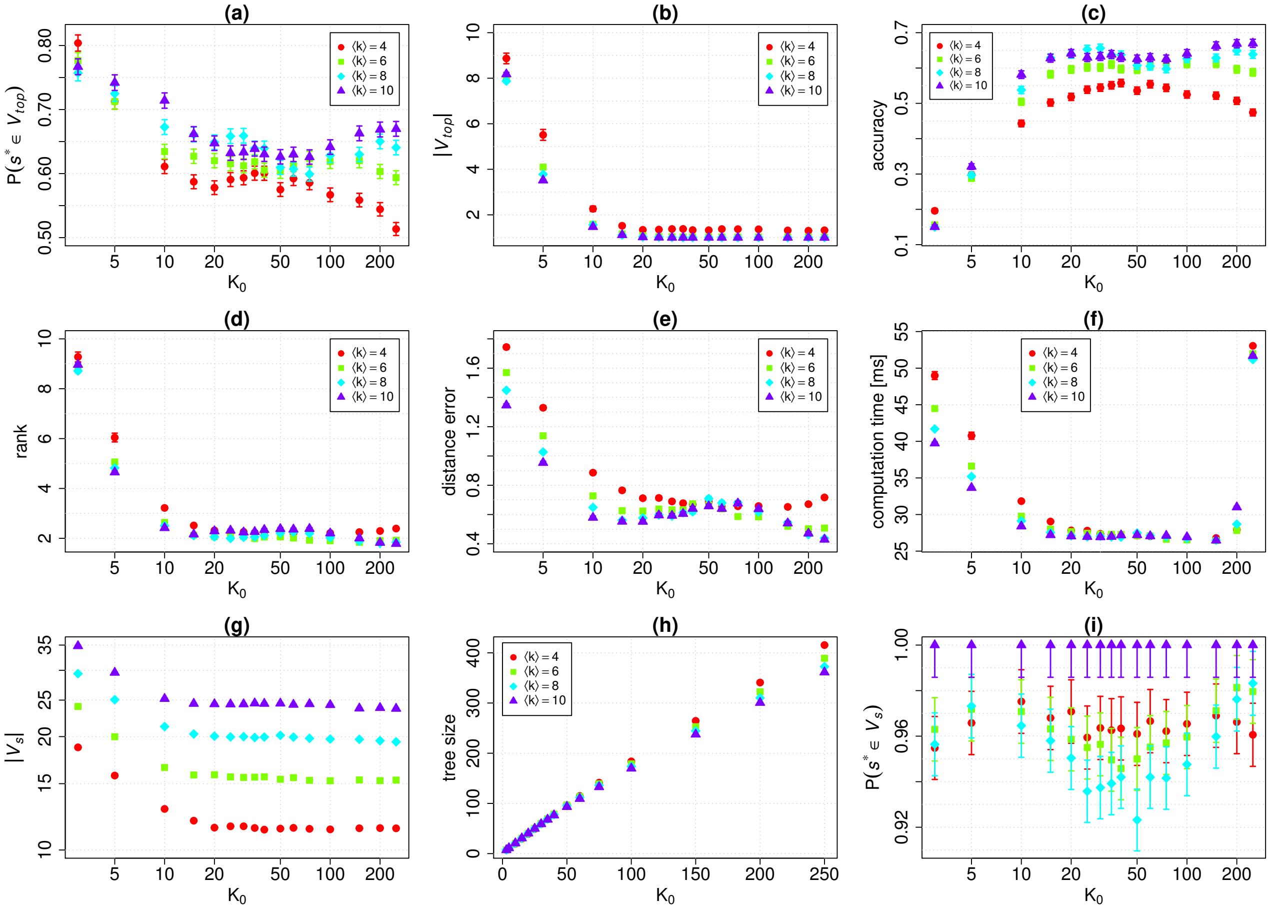

Figure S16: Performance of GMLA versus the number of the nearest observers K_0 for ER network with $N = 1000$ and $\langle k \rangle \in \{4, 6, 8, 10\}$. The observers were distributed randomly over whole network with density $\rho = 0.3$. SI model was used for propagation with infection rate $\beta = 0.5$ which gives propagation ratio $\lambda = \sqrt{2}$. Each point is the result of 5000 realizations. (a) Detection rate. The empirical probability that the true source is in a group of top scorers. (b) Mean size of the group of top scorers. (c) Accuracy. (d) Mean rank of the true source. It takes into account only cases when $s^* \in V_s$. (e) Mean distance error. (f) Computation time. (g) Mean number of suspected nodes $N_0 = |V_s|$ for which scores were determined. (h) Mean size of the propagation tree created for each suspected node. (i) The empirical probability that the true source is in a group of suspected nodes (and has calculated score).

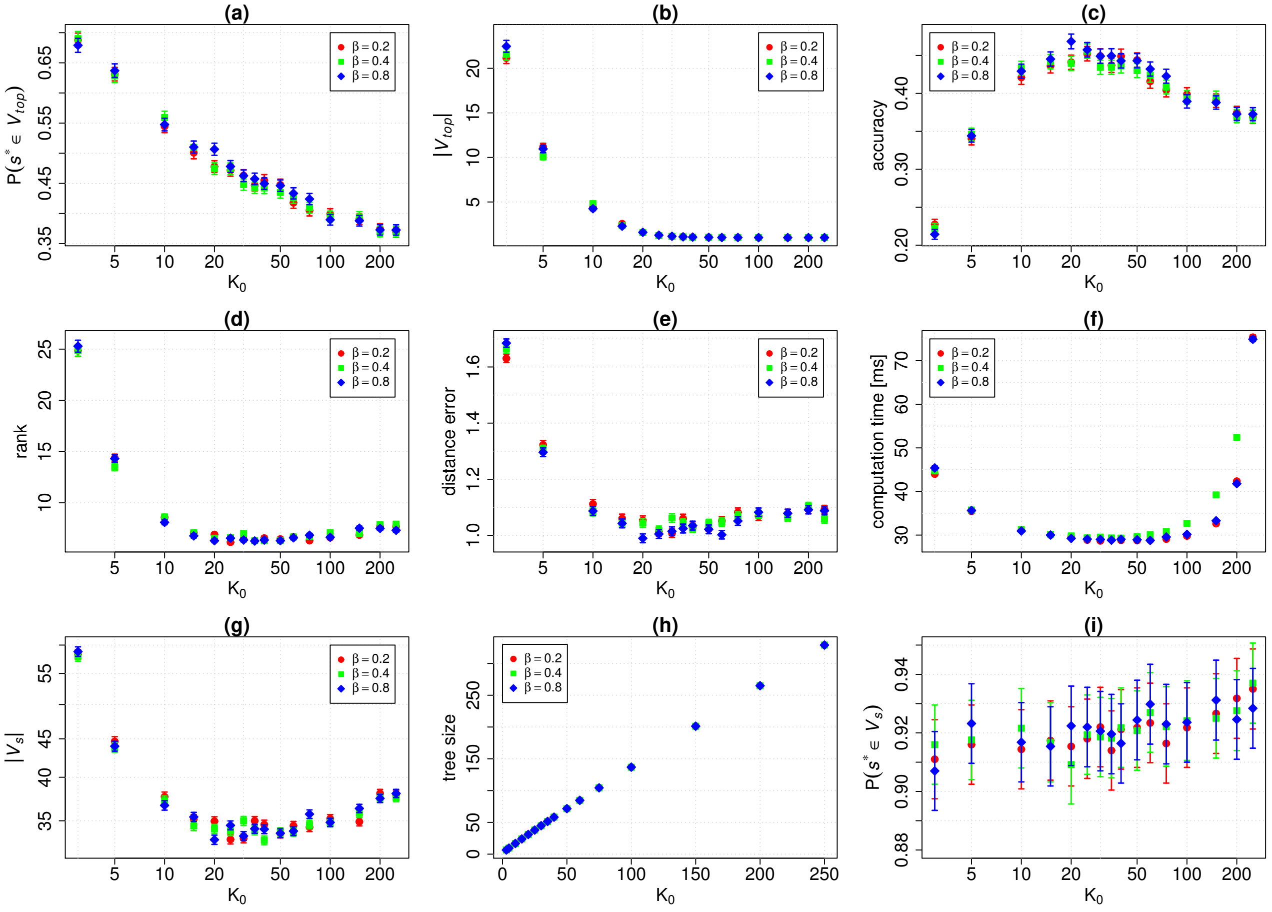

Figure S17: Performance of GMLA versus the number of the nearest observers K_0 for BA network with $N = 1000$ and $m = 3$. The observers were distributed randomly over whole network with density $\rho = 0.3$. SI model was used for propagation with infection rate $\beta \in \{0.2, 0.5, 0.8\}$ which gives propagation ratio $\lambda = \{\sqrt{5}/2, \sqrt{2}, \sqrt{5}\}$. Each point is the result of 5000 realizations. (a) Detection rate. The empirical probability that the true source is in a group of top scorers. (b) Mean size of the group of top scorers. (c) Accuracy. (d) Mean rank of the true source. It includes only the cases when $s^* \in V_s$. (e) Mean distance error. (f) Computation time. (g) Mean number of suspected nodes $N_0 = |V_s|$ for which scores were determined. (h) Mean size of the propagation tree created for each suspected node. (i) The empirical probability that the true source is in a group of suspected nodes (and has calculated score).

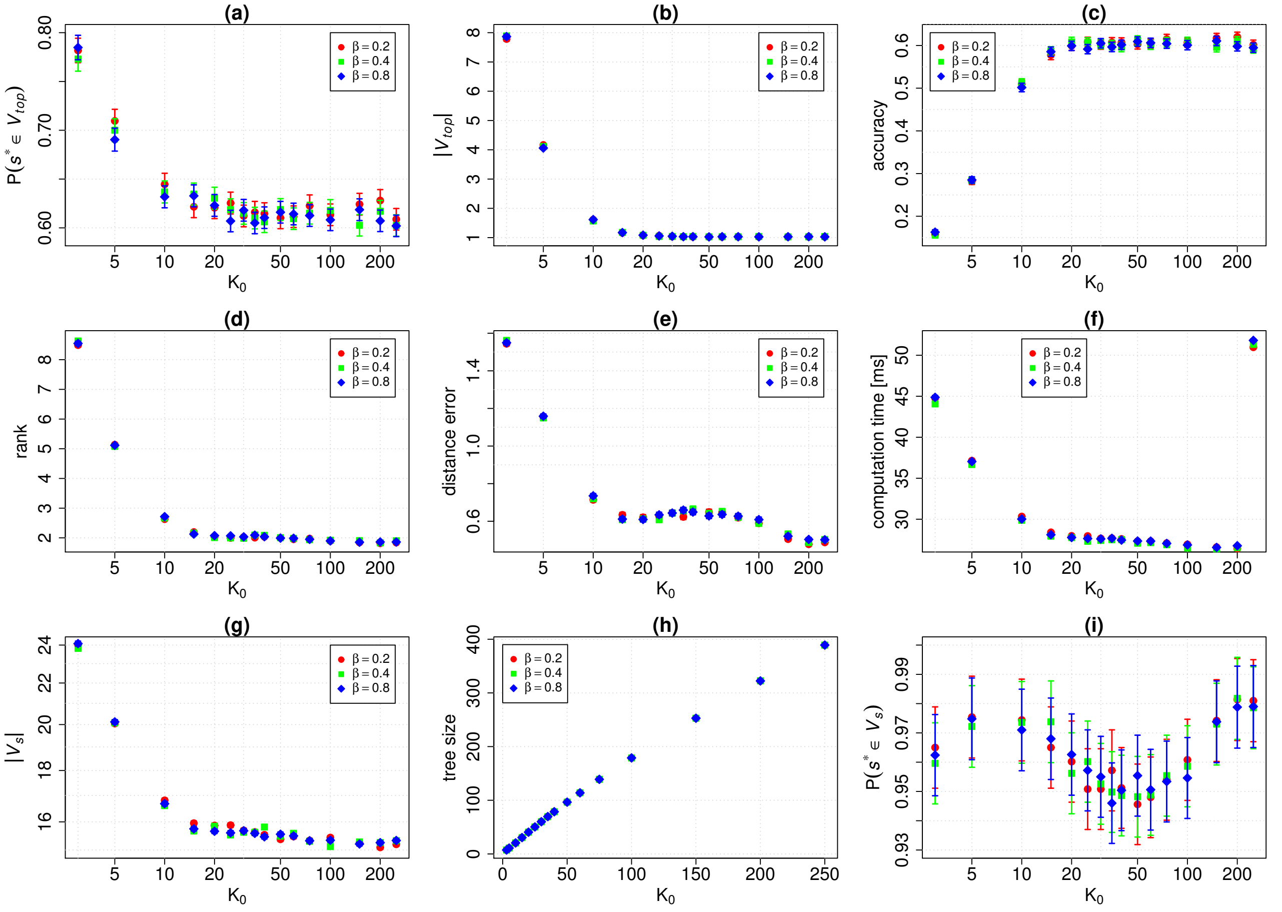

Figure S18: Performance of GMLA versus the number of the nearest observers K_0 for ER network with $N = 1000$ and $\langle k \rangle = 6$. The observers were distributed randomly over whole network with density $\rho = 0.3$. SI model was used for propagation with infection rate $\beta \in \{0.2, 0.5, 0.8\}$ which gives propagation ratio $\lambda = \{\sqrt{5}/2, \sqrt{2}, \sqrt{5}\}$. Each point is the result of 5000 realizations. (a) Detection rate. The empirical probability that the true source is in a group of top scorers. (b) Mean size of the group of top scorers. (c) Accuracy. (d) Mean rank of the true source. It includes only the cases when $s^* \in V_s$. (e) Mean distance error. (f) Computation time. (g) Mean number of suspected nodes $N_0 = |V_s|$ for which scores were determined. (h) Mean size of the propagation tree created for each suspected node. (i) The empirical probability that the true source is in a group of suspected nodes (and has calculated score).

S.4 Performance of GMLA for various networks

In addition to the tests performed on Erdős-Rényi and Barabási-Albert networks (Fig. 3-4 in the main paper), we present the results for Random Regular Graphs, Exponential Random Graphs (in two versions, generated from the configuration model and evolved by random node attachment) and scale-free networks with degree distribution $P(k) \sim k^{-3}$ (created using the configuration model). Both algorithms achieve best results for Random Regular Graphs (Fig. S19), but PTVA-LI performs better than GMLA, except for the computation time. For Exponential Random Graphs the algorithms have very similar quality of the source localization with a slight advantage of PTVA-LI for the configuration model (Fig. S20) and a small advantage of GMLA for the evolving networks S21). Both algorithms have the lowest performance for scale-free networks, but in this case GMLA outperforms substantially PTVA-LI. Deterioration of the quality of detection is the result of existence of hubs in the network (see Discussion in the main paper). The observers which are "behind the hubs" are not reliable (we call them "noisy observers") and distort the estimation of the scores. GMLA uses only K_0 nearest observers, among which there is a lower fraction of the noisy observers than among all observers (Fig. 6 in the main paper). This in combination with the gradient-like selection of suspected nodes improves the quality of the algorithm's results.

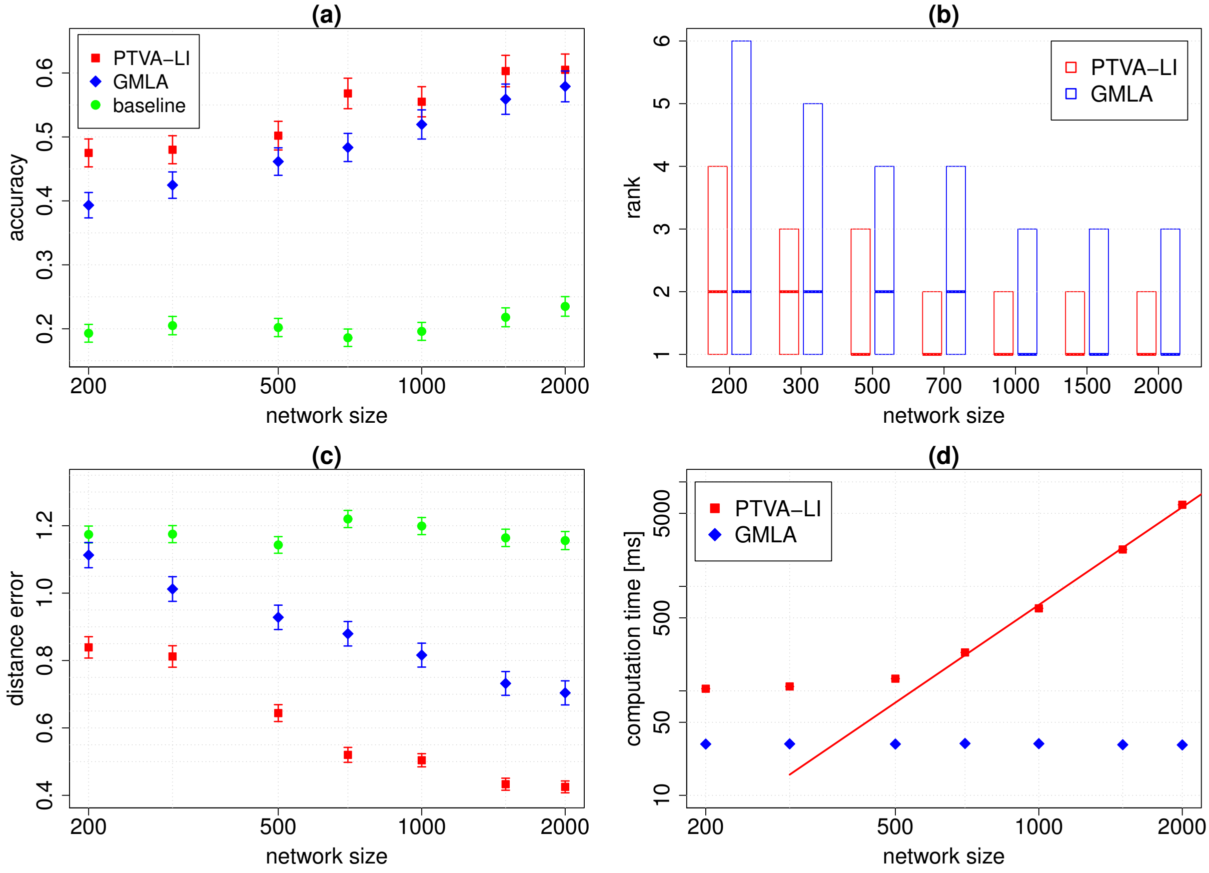

Figure S19: GMLA (blue) versus PTVA-LI (red) and the baseline method (green) on Random Regular Graphs ($k = 6$). (a) Accuracy of both algorithms increases with the network size, but PTVA-LI performs slightly better. (b) The band inside the box shows the median of the rank of the true source. The bottom and top of the box show values of the first and third quartiles. PTVA-LI has lower rank than GMLA. (c) The mean distance error of both algorithms decreases with the network size, but PTVA-LI performs better. (d) The mean computation time of a single realization of GMLA is substantially smaller that PTVA-LI. The solid line is a power law with exponent 3.1 (red). Parameters: $k = 6$, $\lambda = \sqrt{2}$, $\rho = 0.2$, $K_0 = 0.5\sqrt{N}$.

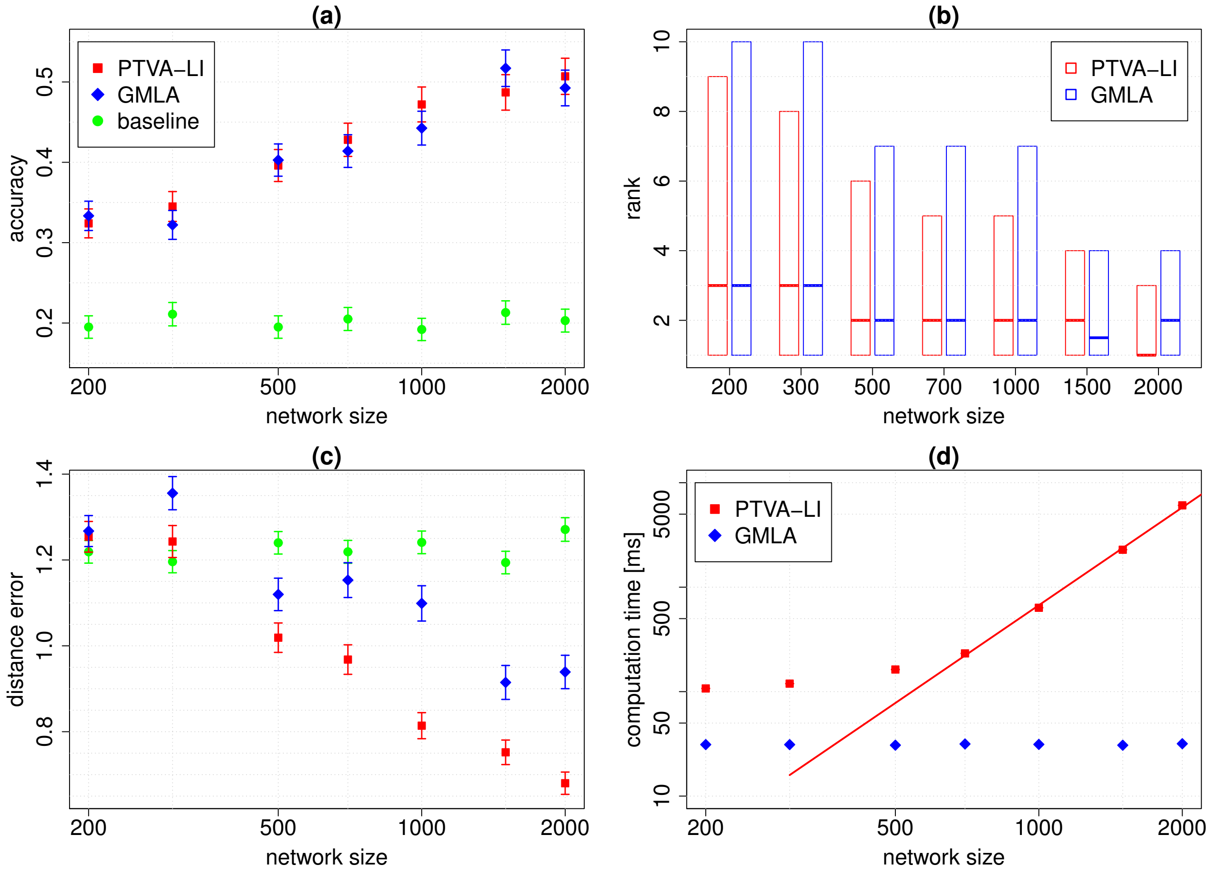

Figure S20: GMLA (blue) versus PTVA-LI (red) and the baseline method (green) on Exponential Random Graphs (created using the configuration model). (a) Accuracy of both algorithms is similar and increases with the network size. (b) The band inside the box shows the median of the rank of the true source. The bottom and top of the box show values of the first and third quartiles. PTVA-LI has slightly lower rank than GMLA. (c) The mean distance error of both algorithms decreases with the network size, but PTVA-LI performs better. (d) The mean computation time of a single realization of GMLA is substantially smaller that PTVA-LI. The solid line is a power law with exponent 3.1 (red). Parameters: $k = 6$, $\lambda = \sqrt{2}$, $\rho = 0.2$, $K_0 = 0.5\sqrt{N}$.

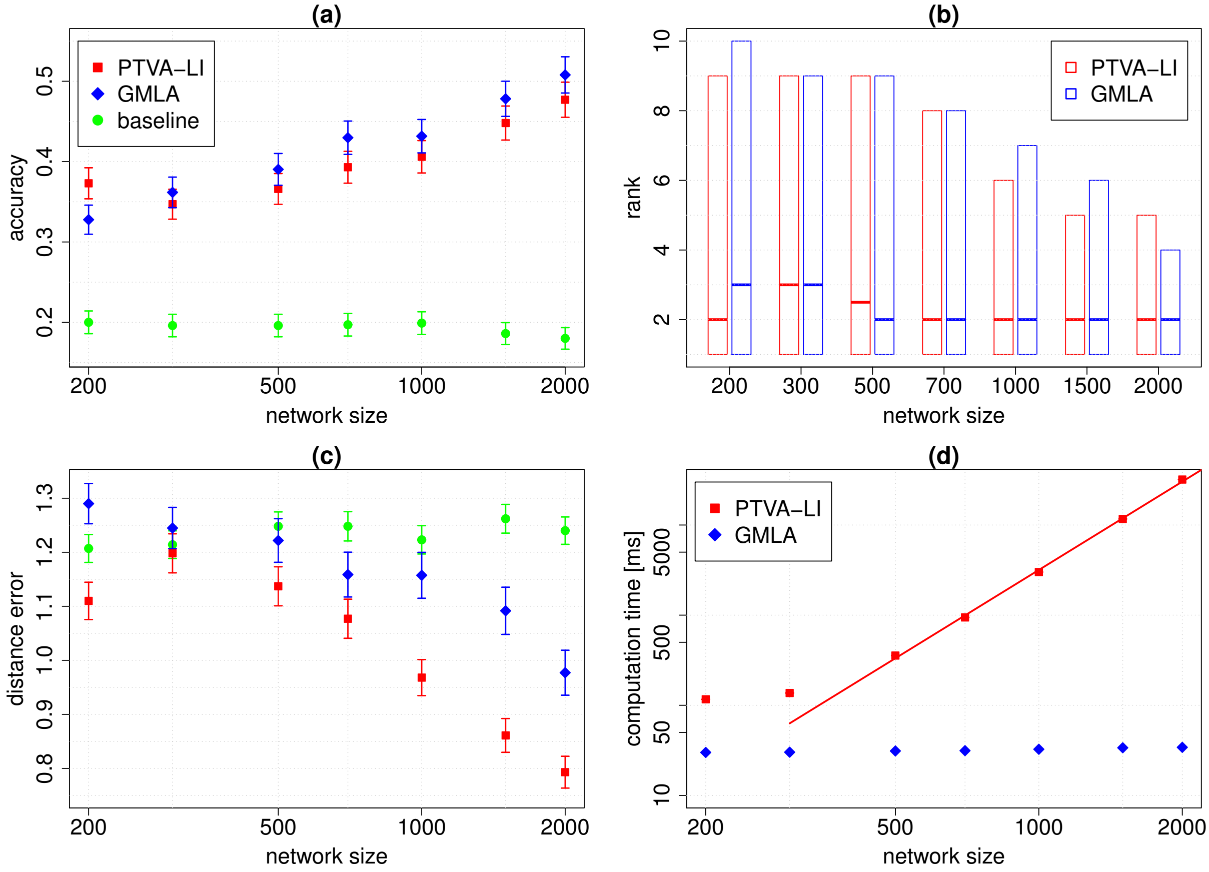

Figure S21: GMLA (blue) versus PTVA-LI (red) and the baseline method (green) on Exponential Random Graphs (evolved by random node attachment). (a) Accuracy of both algorithms increases with the network size, but GMLA performs slightly better. (b) The band inside the box shows the median of the rank of the true source. The bottom and top of the box show values of the first and third quartiles. Results of both algorithms have similar rank. (c) The mean distance error of both algorithms decreases with the network size, but PTVA-LI performs better. (d) The mean computation time of a single realization of GMLA is substantially smaller that PTVA-LI. The solid line is a power law with exponent 3.3 (red). Parameters: $k = 6$, $\lambda = \sqrt{2}$, $\rho = 0.2$, $K_0 = 0.5\sqrt{N}$.

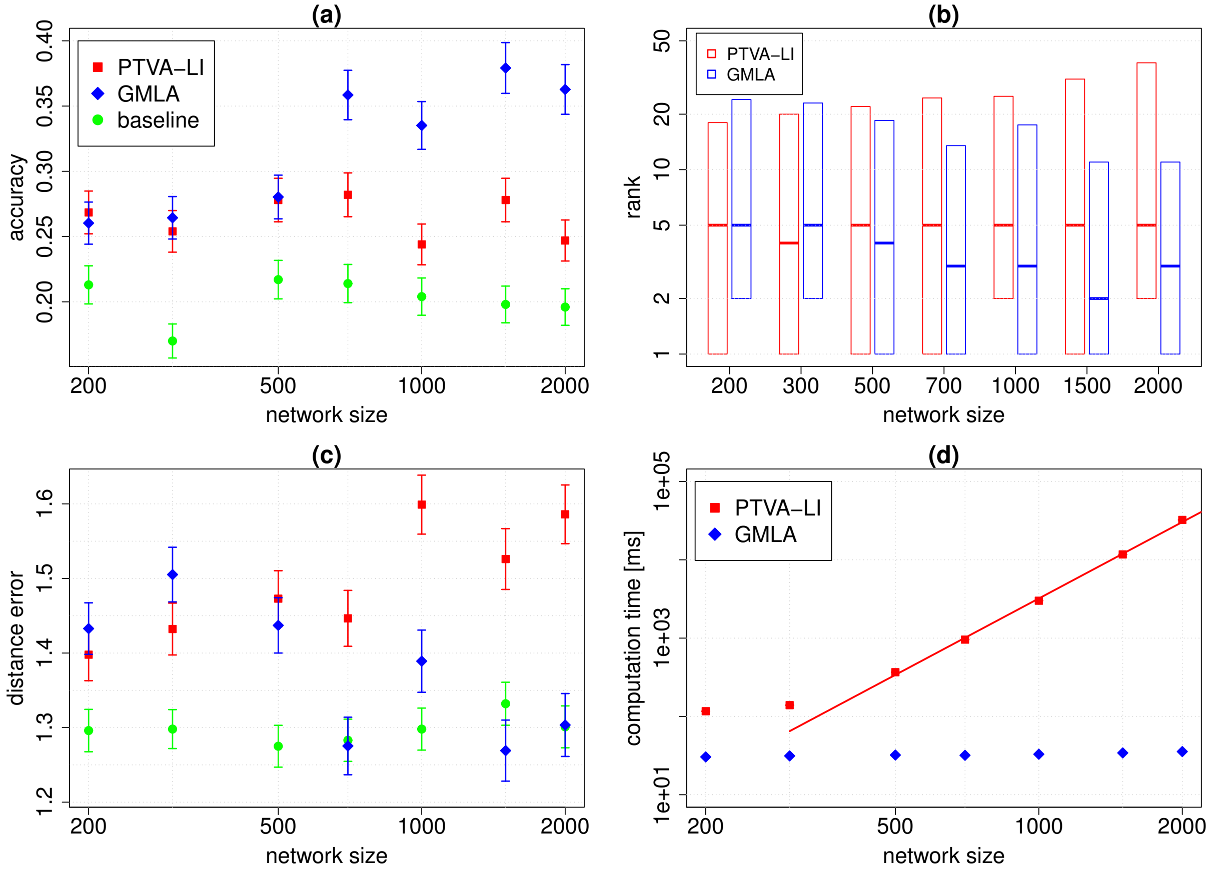

Figure S22: GMLA (blue) versus PTVA-LI (red) and the baseline method (green) on scale-free networks with degree distribution $P(k) \sim k^{-3}$ (created using the configuration model). (a) Accuracy of GMLA increases with the network size and it is much higher than accuracy of PTVA-LI. (b) The band inside the box shows the median of the rank of the true source. The bottom and top of the box show values of the first and third quartiles. GMLA has noticeably lower rank than PTVA-LI. (c) The mean distance error for PTVA-LI increases with the network size in contrast to GMLA, which performs better than PTVA-LI for networks with $N = 500$ nodes or bigger. (d) The mean computation time of a single realization of GMLA is substantially smaller that PTVA-LI. The solid line is a power law with exponent 3.2 (red). Parameters: $\langle k \rangle = 6$, $\lambda = \sqrt{2}$, $\rho = 0.2$, $K_0 = 0.5\sqrt{N}$.
